# The effect of intrapartum maternal fever on neonatal outcomes: a systematic review and meta-analysis

**DOI:** 10.3389/fped.2025.1571732

**Published:** 2025-09-17

**Authors:** Qian Ling, Haixia Wan

**Affiliations:** Department of Gynecology and Obstetrics, Huzhou Maternity & Child Health Care Hospital, Huzhou, Zhejiang, China

**Keywords:** intrapartum fever, neonatal outcomes, maternal fever, term pregnancy, singleton pregnancy, chorioamnionitis, meta-analysis vaginal

## Abstract

**Objective:**

To systematically review the link between intrapartum maternal fever and adverse neonatal outcomes in term singleton pregnancies not complicated by chorioamnionitis.

**Methods:**

The PubMed, Web of Science, Scopus, and Embase databases were searched for studies published up to June 30, 2024, that reported data on women with term singleton pregnancies and intrapartum fever. Studies describing cases of chorioamnionitis (CAM) were excluded. The included studies had to have defined exclusion criteria to ensure that women with a high likelihood of CAM were excluded. Neonatal outcomes of interest were infection/sepsis, fetal distress, assisted ventilation, low APGAR scores, neonatal intensive care unit (NICU) admission, seizures, and hypotonia. Study quality was assessed by the Newcastle-Ottawa Scale (NOS). A random-effects model was used to pool effect sizes, which were reported as odds ratios (OR) and weighted mean differences (WMD). Funnel plots and Egger's test were used to assess publication bias.

**Results:**

A total of 11 studies (*n* = 153,410) were included. Neonates born to mothers with intrapartum fever had a higher risk of low APGAR scores (OR 2.97, 95% CI: 1.61, 5.48), need for assisted ventilation (OR 2.50, 95% CI: 1.59, 3.93), infection/sepsis (OR 6.01, 95% CI: 2.68, 13.5), NICU admission (OR 2.77, 95% CI: 1.40, 5.51), seizures (OR 4.25, 95% CI: 1.95, 9.22), and hypotonia (OR 4.19, 95% CI: 1.72, 10.2). The birth weight of neonates delivered by febrile mothers was significantly higher (WMD 63.4 g, 95% CI: 16.2, 110.5). Publication bias was noted for low APGAR scores and neonatal infection/sepsis.

**Conclusion:**

Intrapartum maternal fever appears to be associated with increased risks of adverse neonatal outcomes. However, the challenge of entirely excluding CAM-related fever and variability in study methodologies limits the robustness of the findings. Nonetheless, proactive management of maternal fever during labor could be critical.

**Systematic Review Registration:**

https://www.crd.york.ac.uk/PROSPERO/view/CRD42024565830, PROSPERO CRD42024565830.

## Introduction

Intrapartum maternal fever, which refers to a body temperature of 38°C (100.4°F) or higher during labor, is relatively common, with a prevalence of around 2–10% of all pregnancies (1, 2). Causes of intrapartum fever may include infection as well as the response of the body to labor itself (2–4). Furthermore, studies show the link between epidural analgesia and the occurrence of intrapartum fever with possible adverse outcomes both in women and in offspring (5–8). The use of misoprostol, a commonly employed induction agent, is another potential cause of intrapartum fever (9, 10).

In preterm gestation, intrapartum fever often serves as a critical indicator of intra-amniotic infection and is linked to poorer neonatal outcomes (11, 12). However, the implications of maternal fever in term pregnancies are less clear. While several studies report adverse neonatal outcomes in term pregnancies complicated by fever, most cases of intrapartum fever in these studies are due to chorioamnionitis (CAM) (13, 14).

The objective of this meta-analysis is to systematically review and synthesize the existing evidence on the intrapartum fever-associated risk of adverse outcomes in full-term neonates who were born to mothers with no intra-amniotic infection/CAM.

## Methodology

### Search for potential studies

A systematic literature search of PubMed, Web of Science, Cochrane library, Chinese National Knowledge Infrastructure (CNKI), Scopus, and Embase databases was done for papers published until 30th June 2024. The search strategy for each of the databases is provided in the Supplementary Appendix. The study adhered to the PRISMA guidelines (15), and the protocol was registered before the commencement of the review (PROSPERO; https://www.crd.york.ac.uk/prospero/) (CRD42024565830).

### Inclusion and exclusion criteria

This review focused on women experiencing intrapartum fever during term singleton pregnancies without the presence of chorioamnionitis (CAM). All included studies had defined exclusion criteria to ensure that women with a high likelihood of CAM were not included. Ideally, only studies where a confirmed diagnosis of CAM was explicitly ruled out were to be included. However, this information was not available in most of the potential studies. As a result, the inclusion criteria were adjusted to be more flexible to accommodate the available studies. Specifically, studies were required to exclude women with fever at the time of admission, those with acute inflammatory conditions such as genital or upper respiratory tract infections, and those without documented fever measurements. Additionally, studies that did not include women with placental pathology indicative of CAM were considered. Studies where women were diagnosed with clinical CAM, i.e., fever accompanied by at least two of the following signs: fetal or maternal tachycardia, leukocytosis, uterine tenderness, or foul-smelling discharge, were also excluded. Furthermore, studies that included women who had received prostaglandins during labor induction or those with ruptured membranes for more than 24 h were excluded.

Studies needed to report on at least one neonatal outcome, including neonatal infection/sepsis, respiratory distress, need for assisted ventilation, low Apgar scores, admission to the neonatal intensive care unit (NICU), and neonatal morbidity. The review included cohort (both prospective and retrospective) and case-control studies that provided quantitative measures linking intrapartum maternal fever to adverse neonatal outcomes. Reviews, meta-analyses, conference abstracts, case reports, and editorials were excluded to maintain focus on primary research findings. Studies lacking adequate control groups or failing to differentiate between term and preterm gestations were also excluded to ensure the relevance and clarity of the findings.

### Process of selecting the final set of studies and data extraction

After establishing the initial pool of relevant studies through database searches, duplicate articles were removed. Subsequently, titles and abstracts were screened for studies that aligned with the research objectives. Full-text reviews were then performed on potentially relevant studies, applying additional exclusion criteria as needed to comprise a final pool of studies. Two authors independently carried out each stage of the process. All differences were resolved through consensus.

Two authors independently used a structured data extraction to retrieve critical study details, including author, publication year, study location, and design, the definition of intrapartum fever, sample size, type of delivery, and key outcomes assessed. All differences at that stage were resolved through consensus.

### Quality assessment and statistical analysis

The Newcastle-Ottawa Scale (NOS) was used for quality assessment. The NOS score has a maximum score of 9, with a higher score indicating better study quality (16). Methodological aspects such as selection of study groups, comparability, and ascertainment of outcomes were assessed (16). The pooled effect sizes were reported as odds ratios (OR) for categorical outcomes and weighted mean differences (WMD) for continuous outcomes, along with 95% confidence intervals (CIs). A random-effects model was used for the analysis to account for potential variability across the included studies (17). An additional exploration was conducted to understand the association between maternal fever duration and the outcomes of interest. Only four studies reported some findings related to the duration of maternal fever and maternal and/or neonatal outcomes. These reported outcomes varied across these four studies, and therefore, pooled estimates were not generated. Heterogeneity assessment was done using the *I*^2^ statistic. Subgroup analysis and sensitivity analysis were performed to explore the source of heterogeneity. The individual findings of these studies were systematically documented. Funnel plots and Egger's test assessed publication bias (18). *P* < 0.05 was considered to denote statistical significance. All statistical analyses were performed using STATA version 15.0.

## Results

### Search results

**Figure 1:**
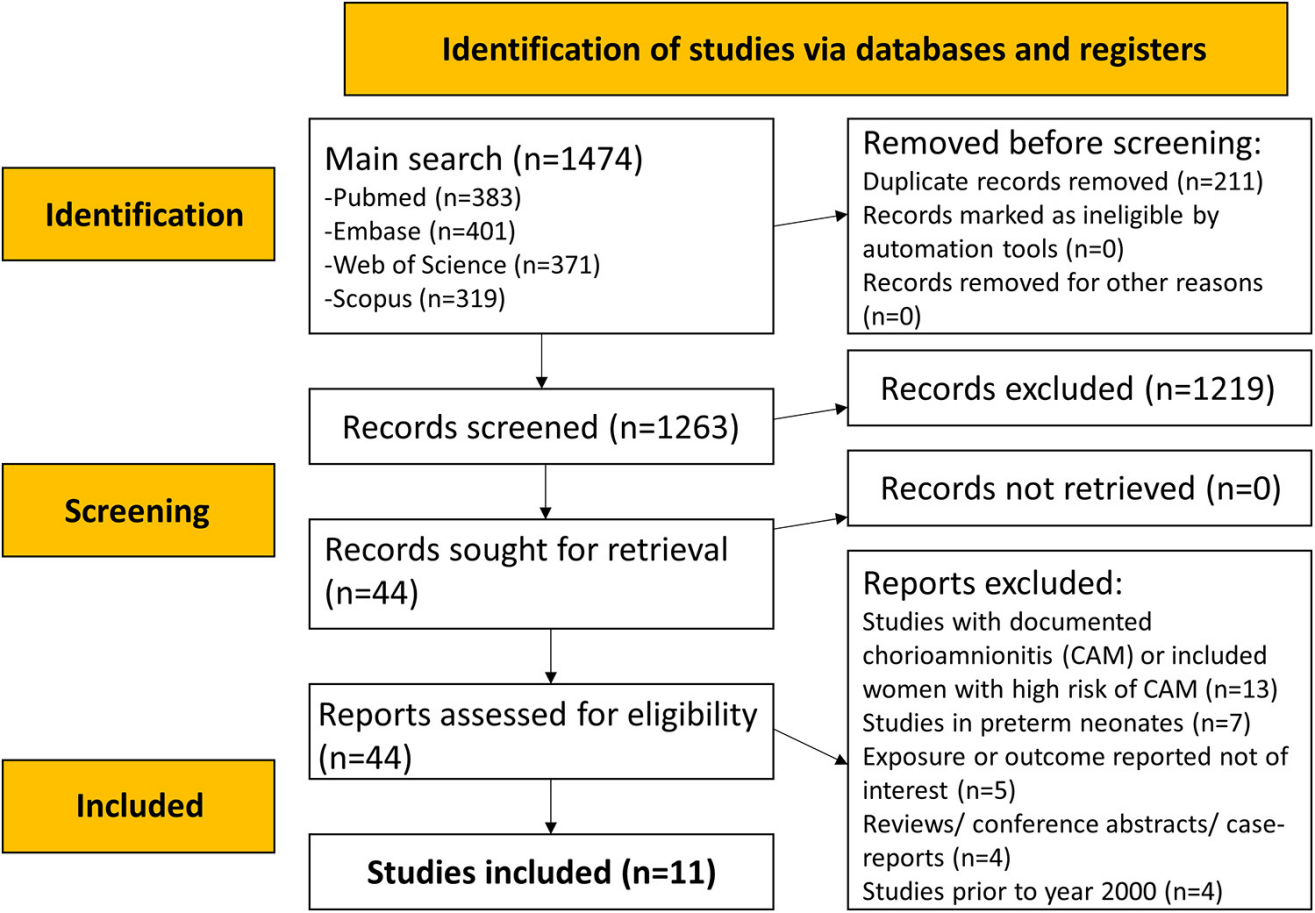
PRISMA flowchart to show the process of study selection.

The literature search identified a total of 1,474 studies. After eliminating 211 duplicates, titles and abstracts of 1,263 studies were searched (Figure 1), and an additional 1,219 studies were excluded. The full texts of 44 papers were reviewed. Ultimately, 11 eligible studies were included in the meta-analysis (Figure 1) (3, 19–28).

### Characteristics of the included studies

As shown in Table 1, most studies had a retrospective cohort design (*n* = 9). One study employed a case-control design, and another was a prospective cohort study (*n* = 1). Studies were done in China (*n* = 3), the United States (*n* = 3), Israel (*n* = 4), and Canada (*n* = 1). The included studies varied in their definitions of intrapartum fever. Four studies defined fever as a temperature of ≥38°C, three studies used >38°C, two studies used ≥37.5°C, and one study each used >37.8°C as a criterion for the diagnosis of intrapartum fever. Vaginal delivery was predominantly reported in almost all the included studies. The total sample of all studies included 153,410 women. Of them, 4,179 were diagnosed with fever, and 149,231 were without fever. All included studies were of modest quality, as indicated by a mean NOS score of 6.91 (Table 2).

**Table 1:** Included studies with their brief characteristics.

Author	Study design; location	Definition of intrapartum fever	Sample size	Exclusion criteria relevant to the research question	Analysis by the duration of intrapartum fever	Type of delivery	Key outcomes assessed
Zhang et al. (2023) (19)	PC; China	Highest axillary temperature during labor more than or equal to 37.5°C.	Fever (74) No fever (503)	Excluded clinically assessed “high-risk” pregnancies. No definitive assessment of CAM	Data on duration not provided	Vaginal (66%)	Foetal distress Low APGAR (5 min) Need for Assisted Ventilation Neonatal infection/sepsis Admission to NICU Birth weight (grams)
Wang et al. (2023) (20)	RC; China	Highest axillary temperature during labor more than or equal to 37.5°C.	Fever (42) No fever (166)	Exclude those with pre-epidural temperature of ≥37.5°C; taking paracetamol within 6 h of epidural; with pre-epidural acute inflammatory diseases (e.g., genital tract or acute upper respiratory tract infections	Data on duration not provided	Vaginal (100%)	Foetal distress Neonatal infection/sepsis Admission to NICU Birth weight (grams)
Hochler et al. (2021) (21)	RC; Israel	Defined as a temperature of ≥38.0°C (≥100.4°F), obtained by an oral measurement	Fever (1,517) No fever (84,196)	Those with no documentation of fever measurement or those presenting with fever on admission were excluded; Upon fever- blood/vaginal/urine cultures were done	Data on duration provided and findings presented	Vaginal (87%)	Need for Assisted Ventilation Low APGAR (5 min) Admission to NICU Neonatal infection/sepsis Seizure Birth weight (grams)
Ren et al. (2021) (22)	RC; China	Defined as a maximum temperature >37.5°C, measured using probe in external auditory canal	Fever (495) No fever (1,556)	Exclusion was based on: baseline body temperature in the delivery room higher than 37.5°C; lower genital tract infection or upper respiratory tract infection; placental pathology examination indicating a diagnosis of chorioamnionitis	Data on duration not provided	Vaginal (100%)	Admission to NICU Low APGAR (5 min)
Ashwal et al. (2018) (23)	RC; Israel	Defined as at least one temperature measurement of ≥38.0°C (100.4°F)	Fever (309)No fever (618)	Women with orally measured temperature of ≥37.5°C (99.5°F) upon admission were excluded; Upon fever- blood/vaginal/urine cultures were done and placental swabs taken after delivery	Data on duration provided and findings presented	Vaginal (88%)	Low APGAR (5 min) Need for Assisted Ventilation Seizure Birth weight (grams)
Burgess et al. (2017) (3)	RC; USA	Highest temperature during labor more than 38°C	Fever (54) No fever (306)	Excluded if a diagnosis of clinical chorioamnionitis made, i.e., presence of fever accompanied by at least 2 of the following signs: fetal tachycardia >160 beats per minute, maternal tachycardia >100 beats per minute, maternal leukocytosis (>15,000 cells/mm), uterine tenderness, or foul-smelling vaginal discharge	Data on duration not provided	Vaginal (74%)	Low APGAR (5 min) Admission to NICU Birth weight (grams)
Dior et al. (2016) (24)	RC; Israel	Highest oral temperature during labor between 38.0 and 38.9°C.	Fever (898) No fever (42,601)	Excluded those with documented use of prostaglandins during induction of labor (due to their effect on body temperature elevation)	Data on duration provided and findings presented; No dose response seen between duration of high fever and maternal/neonatal outcomes	Not provided	Low APGAR (5 min) Admission to NICU Neonatal infection/sepsis Seizure
Greenwell et al. (2012) (25)	RC; USA	Maternal fever defined as a maximum intrapartum axillary temperature >100.4°F (≥38.0°C)	Fever (238) No fever (1,538)	Excluded if temperature was never recorded or was >99.5°F at admission; also pregnancies were excluded in which the infant (was later) diagnosed with documented sepsis, meningitis, pneumonia, congenital infections, or viral infections	Data on duration not provided	Vaginal (64%)	Hypotonia Foetal distress Need for Assisted Ventilation Low APGAR (5 min) Seizure Birth weight (grams)
Maayan-Metzger et al. (2006) (26)	CC; Israel	Highest temperature during labor >37.8°C	Fever (330) No fever (330)	Excluded if temperature was >37.8°C at admission	Data on duration not provided	Vaginal (60%)	Birth weight (grams) Need for Assisted Ventilation
Reilly and Oppenheimer (2005) (27)	RC; Canada	Oral maternal temperature >38°C or 2 consecutive temperatures >37.5°C after the onset of active labour	Fever (161) No fever (16,322)	Excluded if women had ruptured membranes for more than 24 h; those presenting with uterine tenderness or foul-smelling amniotic fluid; those with documented presence of chorioamnionitis on placental examination	Data on duration provided; no association of duration of fever with outcomes considered	Vaginal (92%)	Admission to NICU Foetal distress Birth weight (grams)
Lieberman et al. (2000) (28)	RC; USA	Maternal fever defined as intrapartum temperature >101°F (>38.3°C). Most temperatures recorded orally and in case, axillary temperature was recorded, it was increased by 1° Fahrenheit for comparability	Fever (61) No fever (1,095)	Women were excluded if they were diabetic, had an active genital herpes infection, if maternal temperature was never recorded, if a maternal temperature >99.5°F was present at admission, had infants with documented sepsis, pneumonia, or herpes infection	Data on duration not provided	Vaginal (89%)	Need for Assisted Ventilation Hypotonia Admission to NICU Seizure

PC, prospective cohort; RC, retrospective cohort; CC, case-control; NICU, neonatal intensive care unit; CAM, chorioamnionitis.

**Table 2:** Risk of bias assessment.

Author	Selection	Comparability	Outcome	Quality score
Zhang et al. (2023) (19)	3 Points	2 Points	2 Points	7
Wang et al. (2023) (20)	3 Points	2 Points	2 Points	7
Hochler et al. (2021) (21)	3 Points	2 Points	3 Points	8
Ren et al. (2021) (22)	3 Points	2 Points	3 Points	8
Ashwal et al. (2018) (23)	3 Points	2 Points	2 Points	7
Burgess et al. (2017) (3)	2 Points	2 Points	2 Points	6
Dior et al. (2016) (24)	3 Points	2 Points	2 Points	7
Greenwell et al. (2012) (25)	3 Points	2 Points	2 Points	7
Maayan-Metzger et al. (2006) (26)	2 Points	2 Points	2 Points	6
Reilly and Oppenheimer (2005) (27)	3 Points	2 Points	2 Points	7
Lieberman et al. (2000) (28)	2 Points	2 Points	2 Points	6

**Figure 2:**
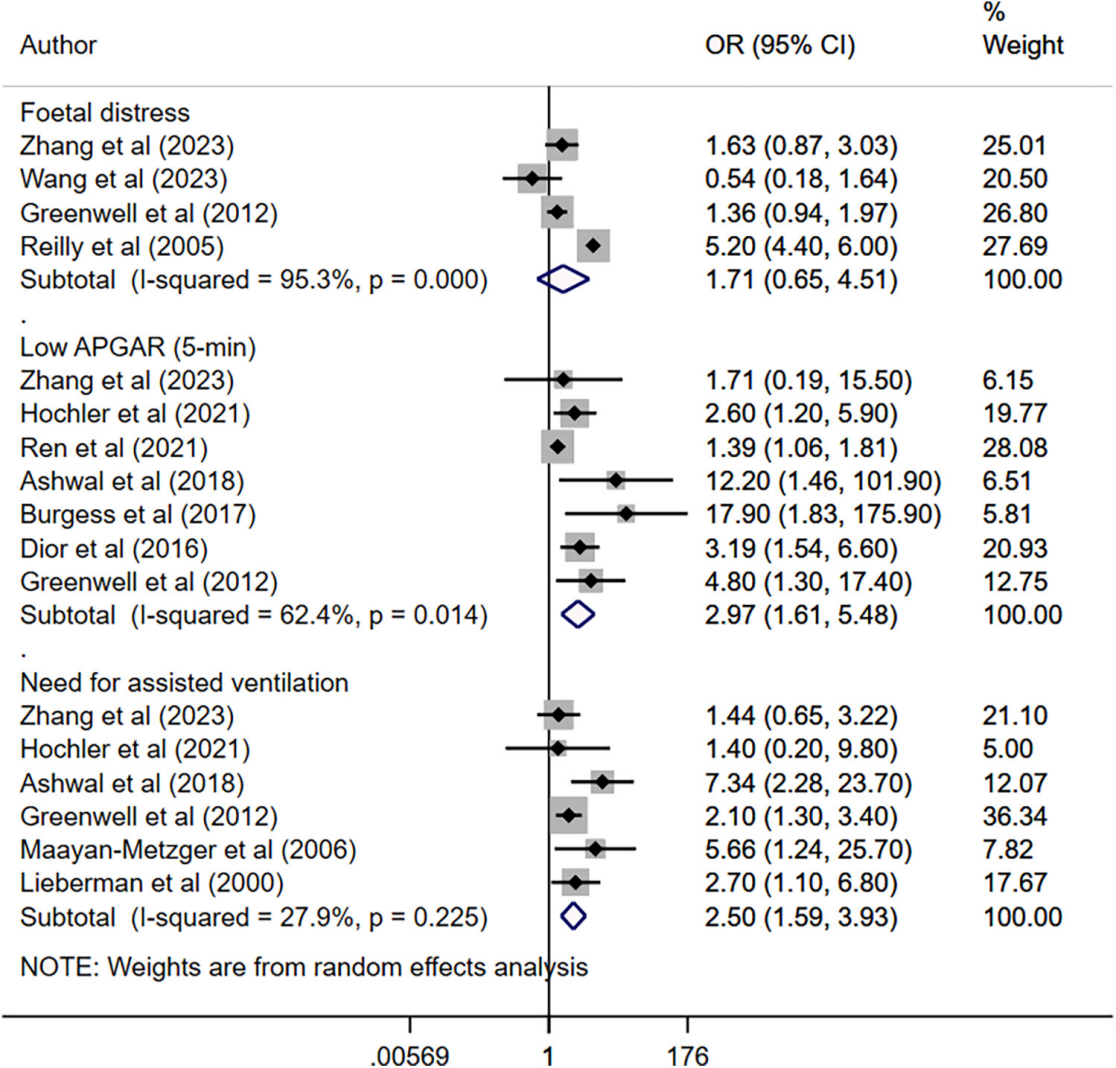
Risk of foetal distress, low APGAR score, and need for assisted ventilation among neonates born to mothers with intrapartum fever, compared to those born to mothers who were afebrile.

**Figure 3:**
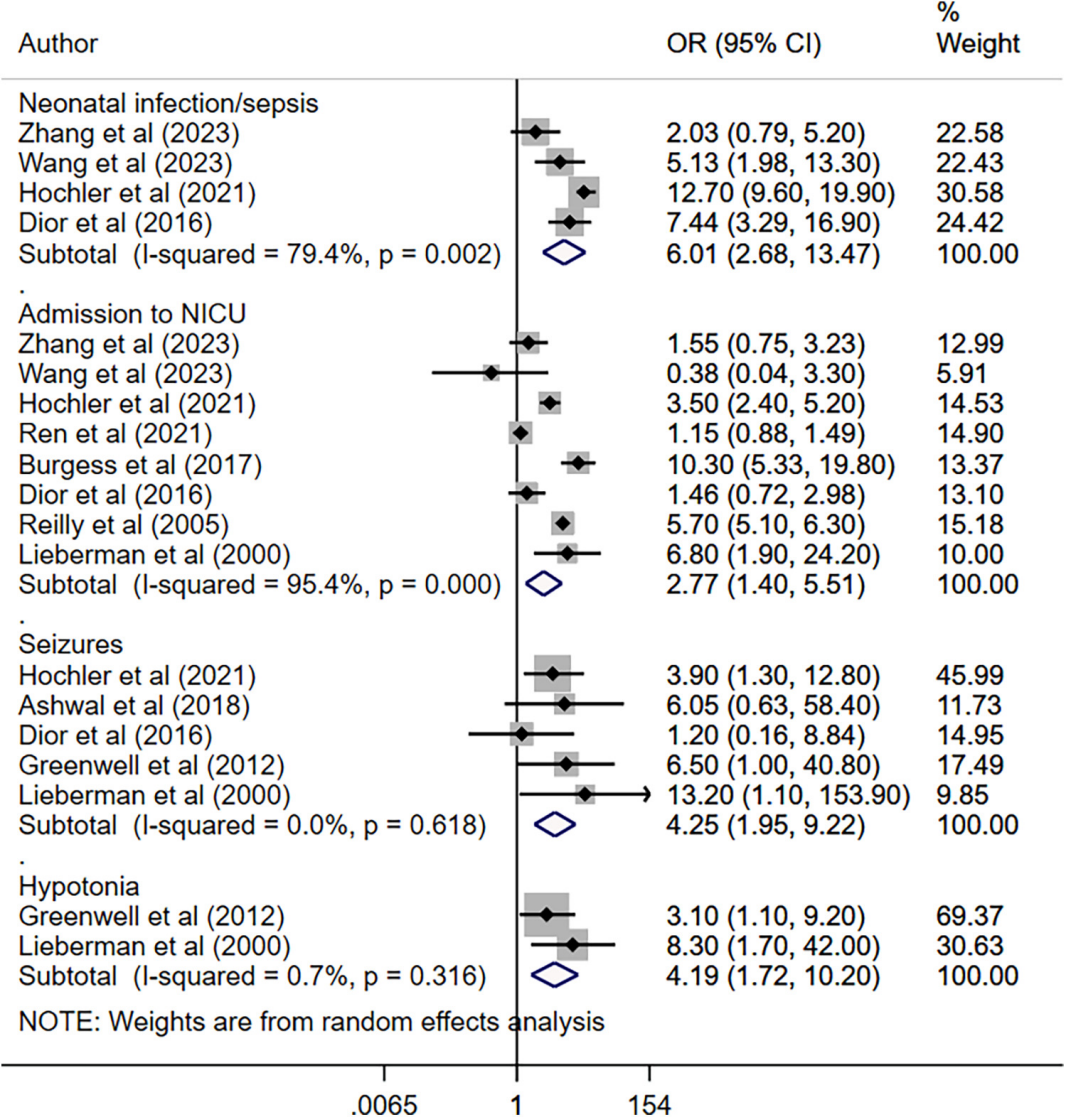
Risk of infection, admission to NICU, seizures, and hypotonia among neonates born to mothers with intrapartum fever, compared to those born to mothers who were afebrile.

**Figure 4:**
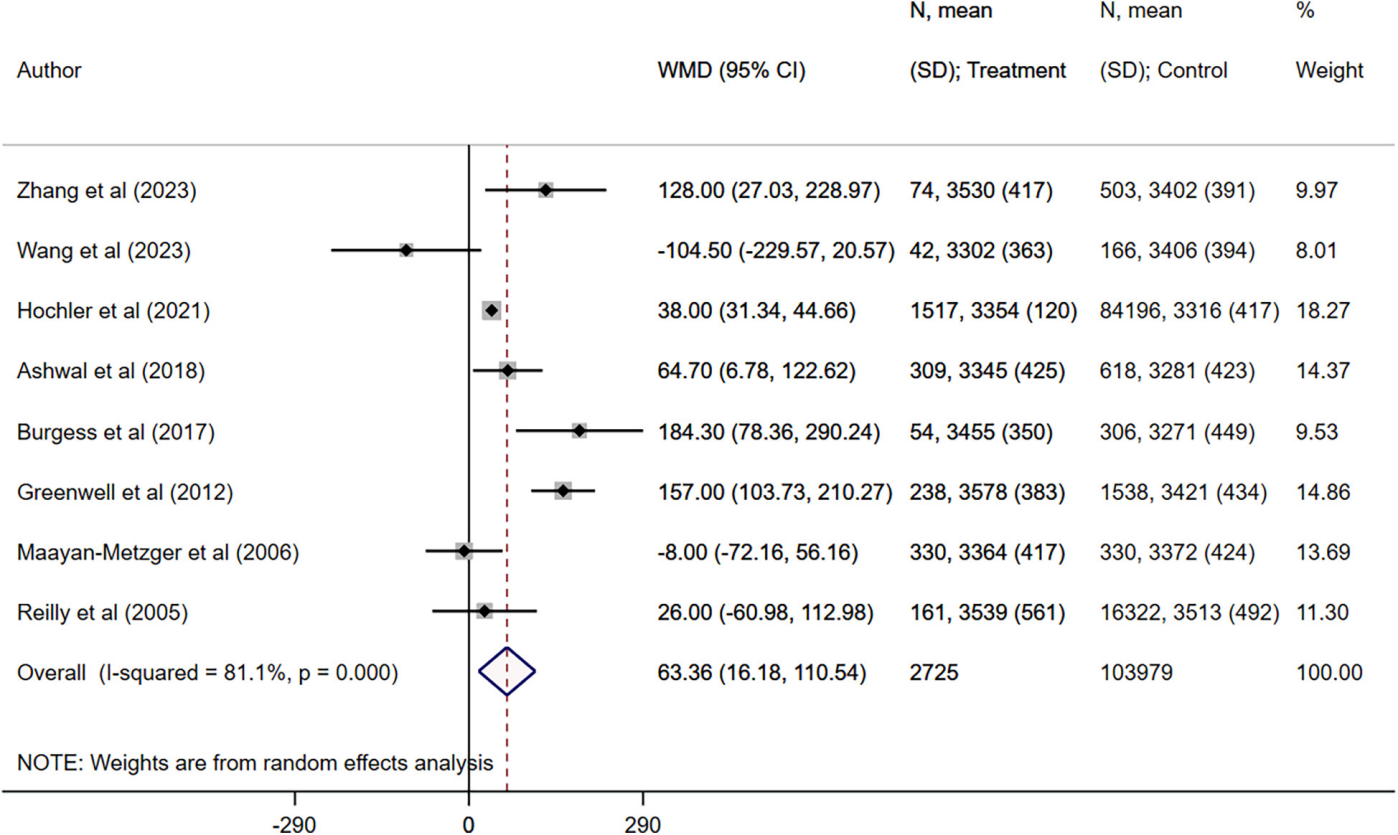
Comparison of birth weight (in grams) between neonates born to mothers with intrapartum fever, compared to those born to mothers who were afebrile.

Maternal fever was associated with a significantly higher risk of having a low APGAR score (OR 2.97, 95% CI: 1.61, 5.48; *n* = 7, *I*^2^ = 62.4%), need for assisted ventilation (OR 2.50, 95% CI: 1.59, 3.93; *n* = 6, *I*^2^ = 27.9%), infection and/or sepsis (OR 6.01, 95% CI: 2.68, 13.5; *n* = 4, *I*^2^ = 79.4%), admission to NICU (OR 2.77, 95% CI: 1.40, 5.51; *n* = 8, *I*^2^ = 95.4%), seizures (OR 4.25, 95% CI: 1.95, 9.22; *n* = 5, *I*^2^ = 0.0%) and hypotonia (OR 4.19, 95% CI: 1.72, 10.2; *n* = 2, *I*^2^ = 0.7%) in offspring (Figures 2, 3). The risk of fetal distress was, however, comparable (OR 1.71, 95% CI: 0.65, 4.51; *n* = 4, *I*^2^ = 95.3%) in neonates born to mothers with or without fever (Figure 2). The birth weight (in grams) of neonates born to febrile mothers was significantly higher (WMD 63.4, 95% CI: 16.2, 110.5; *n* = 8, *I*^2^ = 81.1%) compared to that of neonates born to afebrile mothers (Figure 4). The Egger's test indicated the presence of publication bias for low APGAR score and neonatal infection/sepsis (*p* < 0.05) but not for other outcomes. The funnel plots for each of these outcomes are presented in Supplementary Figures S1–S6. Publication bias for “hypotonia” was not assessed due to the few studies reporting on this outcome.

#### Subgroup analysis

Subgroup analysis for infection and/or sepsis based on fever threshold showed that studies with a threshold of 37.5° had a pooled OR of 3.22 with a 95% CI of 1.30–7.98 (*n* = 2) (Supplementary Figure S7). Studies with a 38-degree threshold had a pooled OR of 11.08 with a 95% CI of 7.01–17.50 (*n* = 2) (Supplementary Figure S8). Subgroup analysis for infection and/or sepsis among studies focusing on vaginal deliveries showed a pooled OR of 3.22 with a 95% CI of 1.30–7.98 (*n* = 2) (Supplementary Figure S9). Analysis based on the type of analgesia was not possible due to the limited number of studies. Subgroup analysis for NICU admission based on fever threshold showed that studies with threshold of 37.5° had pooled OR of 1.17 with 95% CI of 0.92–1.50 (*n* = 3) (Supplementary Figure S10), while studies with 38-degree threshold had pooled OR of 4.51 with 95% CI of 2.73–7.43 (*n* = 5) (Supplementary Figure S11). Subgroup analysis for NICU admission among studies focusing on vaginal deliveries showed a pooled OR of 1.17 with a 95% CI of 0.92–1.50 (*n* = 3) (Supplementary Figure S12). Subgroup analysis for NICU admission among studies focusing on epidural analgesia showed a pooled OR of 1.13 with a 95% CI of 0.87–1.47 (*n* = 2) (Supplementary Figure S13). Subgroup analysis for birth weight (in grams) based on fever threshold showed that studies with threshold of 37.5° had pooled WMD of 69.09 (95% CI: −70.93 to 209.11; *n* = 3) (Supplementary Figure S14), while studies with 38-degree threshold had pooled WMD of 62.58 with 95% CI of 15.78–109.39 (*n* = 4) (Supplementary Figure S15).

#### Sensitivity analysis

Sensitivity analysis for ICU infection and/or sepsis (Supplementary Figure S16), NICU admission (Supplementary Figure S17), and birth weight (Supplementary Figure S18) showed no change in magnitude or direction of association due to potential outliers, indicating the final estimates are robust to single or small study changes.

## Discussion

This meta-analysis suggests that neonates born to mothers experiencing intrapartum fever face increased risks across several critical health outcomes. Notably, maternal fever was associated with significantly increased odds of low APGAR scores, need for assisted ventilation, infection/sepsis, admission to the NICU, seizures, and hypotonia in offspring compared to neonates born to mothers without fever.

The increased risk of low APGAR scores in neonates born to febrile mothers might point towards the immediate physiological impact of intrapartum fever on newborn health. Studies show that intrapartum fever could precipitate fetal stress and compromise oxygenation (29, 30). Such a stress response may manifest as reduced heart rate, respiratory effort, muscle tone, reflex irritability, and pallor, all of which contribute to lower APGAR scores. However, a low APGAR score could also indicate that CAM was not adequately excluded in the studies involved in this review, as it is a direct known outcome of CAM.

Similarly, our observation of the association between maternal fever and higher odds of neonatal infection/sepsis and admission to the NICU reflects the systemic implications of fever on neonatal immune responses. Similar to the finding of low APGAR, it may also mean that CAM was not adequately excluded in the studies that contributed to this outcome. Neonates exposed to maternal intrapartum fever might be more susceptible to early-onset infections, likely due to intrauterine exposure to inflammatory mediators or compromised immune defenses during labor (2, 31, 32). Furthermore, the observed increased odds of seizures and hypotonia highlight potential neurological consequences in neonates. It is plausible that maternal inflammation triggers fever and the release of inflammatory mediators, which may cross the placenta and affect fetal brain development (33, 34). This inflammatory milieu may disrupt normal neuronal activity and increase the susceptibility of neonates to seizures shortly after birth. Additionally, the physiological stress induced by maternal fever could contribute to neonatal hypotonia.

A study by Hochler et al. examined the combined effect of the duration and magnitude of the fever by creating a composite variable. They found that during labor, the risk of adverse neonatal outcomes increased with both higher maternal temperatures and longer fever duration. However, the mode of delivery was not linked to the fever peak or duration (19). In contrast, Dior et al. found no dose-response relationship between fever duration and maternal or neonatal outcomes (22). Similarly, Reilly et al. observed no significant association between fever duration and NICU admission, labour progression, or need for intervention for non-reassuring electronic foetal monitoring (25). Ashwal et al. reported a positive correlation between fever duration and caesarean delivery for labour dystocia, but not with neonatal outcomes (21).

In this study, intrapartum fever was associated with a modest but statistically significant increase in neonatal birth weight (WMD = 63.4 g). While enhanced intrapartum hydration through intravenous fluids likely contributes to transient fetal volume expansion, additional biological mechanisms may also play a role (35). Maternal hyperthermia can elevate basal metabolic rate and uterine blood flow, potentially augmenting placental nutrient delivery and stimulating fetal anabolism (36). Moreover, fever-induced inflammatory mediators such as prostaglandins may alter placental vascular resistance, thereby modulating transplacental transfer of glucose and amino acids (37). It is also possible that increased clinical surveillance of women with intrapartum fever through more frequent ultrasound or Doppler assessments introduces detection bias toward larger-appearing fetuses. Given the heterogeneity of the included studies, these hypothesized pathways remain speculative. Future prospective studies of larger, homogeneous cohorts should measure maternal temperature, fluid balance, and placental perfusion markers alongside neonatal anthropometry to validate and clarify the mechanistic underpinnings of this unexpected weight gain.

Several lines of evidence suggest that maternal interventions and intrapartum management practices may themselves trigger or amplify the febrile response. Notably, administration of oxytocin has been shown to upregulate pro-inflammatory cytokines, including interleukin-6 and tumor necrosis factor-α, through activation of peripheral mononuclear cells, thereby contributing to maternal hyperthermia even in the absence of infection (38). This oxytocin-associated inflammation may cross the placenta, exposing the fetus to elevated cytokine levels that have been implicated in neonatal encephalopathy, dysregulated thermoregulation, and heightened susceptibility to sepsis (39). Similarly, prolonged labor and epidural analgesia have each been linked to rises in maternal core temperature via both infectious (e.g., chorioamnionitis) and non-infectious mechanisms (e.g., decreased heat dissipation), with downstream effects on neonatal acid–base balance and respiratory adaptation (6). Collectively, these data underscore the likelihood that the intensity and duration of maternal fever, not merely its presence, mediate the severity of neonatal complications. However, most existing studies capture only binary fever outcomes or single temperature measurements, limiting our ability to define dose–response relationships between fever burden and neonatal morbidity. Future investigations with a prospective design are needed in which continuous or serial temperature recordings (with standardized thresholds for onset and resolution) are collected alongside biomarkers of inflammation. Such granularity will be essential for unraveling the mechanistic pathways by which intrapartum fever contributes to adverse neonatal outcomes and for informing targeted interventions.

In addition to infectious etiologies, epidural analgesia itself has been implicated in maternal fever through sterile inflammatory mechanisms distinct from pathogen-driven pyrexia (6). Infectious fever is initiated by microbial pyrogens that elevate the hypothalamic set point via prostaglandin-mediated pathways, often accompanied by leukocytosis, elevated C-reactive protein (CRP), and positive amniotic fluid cultures. By contrast, epidural-related fever appears to arise from local cytokine release, particularly interleukin-6 and interleukin-1β at the maternal–fetal interface and within the epidural space, leading to impaired thermoregulatory vasodilation and heat retention rather than systemic infection (6). Clinically, this sterile hyperthermia often presents as a more gradual, low-grade rise in temperature without the typical laboratory or histopathological evidence of chorioamnionitis. Although both fever types can expose the fetus to pro-inflammatory mediators, the absence of microbial invasion in epidural-related cases may reduce the risk of direct neonatal infection. Recognizing these pathophysiological differences is crucial, since infectious fever warrants prompt antibiotic therapy, whereas epidural-related fever may be managed with conservative cooling measures and re-evaluation of analgesic dosing.

This study has some limitations. First, despite the efforts made by the included studies to minimize CAM-related influence by adopting appropriate exclusion criteria, fully isolating non-CAM-related intrapartum fever cases was challenging. It is important to note that most of the included studies were retrospective in design, meaning they relied on pre-existing data. Within these constraints, while the included studies employed the best possible approaches, given the data availability, to exclude cases where intrapartum fever might plausibly be linked to CAM, there is a possibility that CAM may not have been adequately excluded. Second, the heterogeneity in the demographics and methodologies used to define intrapartum fever and assess neonatal outcomes introduces variability in effect sizes. Third, the presence of publication bias, particularly noted for outcomes such as low APGAR scores and neonatal infection/sepsis, suggests the potential for overrepresentation of studies reporting stronger associations. Additionally, limited data were available for some outcomes, such as hypotonia, which restricts the depth of analysis and interpretation for this specific health outcome. Fourth, variability in the quality of included studies, as well as potential confounding factors that were not fully accounted for, may influence the reliability and robustness of the findings. Clinical heterogeneity in fever definition and management protocols across studies further underscores the complexities in synthesizing conclusive evidence. Finally, most of the included studies did not analyze the duration of fever, preventing us from reliably assessing the association between maternal fever duration and adverse maternal or neonatal outcomes. Only four studies provided data on fever duration, and none found a significant association between fever duration and maternal or neonatal outcomes.

Moreover, the predominance of retrospective cohort designs among the included studies imposes important constraints on causal interpretation and confounder control. Key variables such as the use and dosage of labor-inducing agents (e.g., oxytocin, prostaglandins), duration of membrane rupture, epidural analgesia, and maternal comorbidities (including obesity, gestational diabetes, and hypertension) were inconsistently reported and variably adjusted for, if at all. This residual confounding may bias the observed relationships between intrapartum fever and neonatal outcomes, either exaggerating or obscuring true effects. Finally, the analysis was limited by substantial variability in how fever was defined and measured across studies. Thresholds ranged from ≥37.5°C to >38°C, and assessment methods included axillary, oral, and tympanic thermometry without clear calibration standards. Such inconsistencies may have led to differential misclassification of exposure, whereby mild elevations captured by more sensitive cutoffs or measurement sites could be over- or underrepresented in certain cohorts. Consequently, this methodological heterogeneity likely contributes to between-study variance in effect estimates and complicates the interpretation of dose–response relationships.

Finally, there is a risk of bias inherent in the published literature. Publication bias, where studies reporting significant associations between intrapartum fever and neonatal infection or sepsis are more likely to be published in journals, can inflate pooled estimates, as null or negative studies remain unpublished or are buried in grey literature. Within studies, selective outcome reporting and detection bias may further exaggerate effect sizes; for instance, clinicians aware of maternal fever may probe more intensively for neonatal sepsis, leading to differential misclassification. Although small study numbers limited formal funnel-plot and Egger's tests, the possibility of small-study effects cannot be ruled out. Addressing these limitations through standardized methodologies and comprehensive reporting practices will advance our understanding.

### Clinical implications and research directions

The findings of this review and meta-analysis highlight the importance of conducting methodologically rigorous studies that definitively exclude cases of CAM to ensure reliable results. Additionally, future research should consider the duration and intensity of maternal fever and assess its impact on neonatal outcomes. Further investigation into the factors contributing to adverse outcomes in full-term pregnancies without CAM should be a key area of focus.

The association between maternal fever and adverse neonatal outcomes suggests the potential benefit of early detection and management during labor. Routine monitoring of maternal temperature and timely interventions to manage fever and address possible infections may help reduce risks. Prophylactic antibiotics and closer monitoring of neonates born to febrile mothers might be considered to identify any early signs of infection or distress. Additionally, these findings point to the importance of neuroprotective strategies and follow-up care for infants exposed to maternal fever, with early neurological assessments potentially helping to address any long-term developmental concerns.

The included studies had varying definitions of fever to ensure a comprehensive analysis of the available evidence and to avoid excluding studies solely based on the temperature threshold used to define fever. However, this variability in defining fever introduces a degree of heterogeneity that could impact the comparability of findings across studies. A standardized cutoff would enhance consistency and improve the reliability of pooled analyses. Future research should aim to adopt uniform definitions of fever to facilitate more robust comparisons and synthesis of evidence. Longitudinal studies should examine the long-term developmental and health impacts on infants exposed to intrapartum fever, tracking neurological, cognitive, and physical development. Additionally, randomized controlled trials investigating the effectiveness of different fever management strategies during labour will provide evidence-based clinical guidelines.

## Conclusion

This meta-analysis suggests that maternal intrapartum fever might be associated with increased risks of adverse neonatal outcomes. However, the potential influence of chorioamnionitis (CAM), despite efforts to exclude it, cannot be fully ruled out. Variability in the definitions of fever, inclusion criteria, and the retrospective nature of most included studies present notable limitations, introducing potential bias and heterogeneity in the findings. Additionally, the inability to analyze the duration of fever limits insights into its temporal impact on outcomes.

While this study underscores the need for vigilant monitoring and management of maternal fever during labor, it also highlights critical gaps in current research. Future studies should employ standardized fever definitions, more robust methodologies to exclude CAM, and detailed reporting on fever characteristics, including duration. Well-designed studies are crucial for identifying the specific effects of maternal fever and informing targeted interventions. Addressing these research gaps will improve perinatal care and contribute to optimizing neonatal outcomes.

## Data Availability

The original contributions presented in the study are included in the article/supplementary material, further inquiries can be directed to the corresponding author/s.

## Supplementary material

Supplementary Figure S1

Funnel plot for “low APGAR score”.

Supplementary Figure S2

Funnel plot for “need for assisted ventilation”.

Supplementary Figure S3

Funnel plot for “foetal distress”.

Supplementary Figure S4

Funnel plot for “neonatal infection/sepsis”.

Supplementary Figure S5

Funnel plot for “admission to NICU”.

Supplementary Figure S6

Funnel plot for “seizure”.

Supplementary Figure S7

Subgroup analysis of infections and/or sepsis for studies with fever threshold of 37.5 degrees.

Supplementary Figure S8

Subgroup analysis of infections and/or sepsis for studies with fever threshold of 38 degrees.

Supplementary Figure S9

Subgroup analysis of infections and/or sepsis for studies with vaginal delivery.

Supplementary Figure S10

Subgroup analysis of NICU admission for studies with fever threshold of 37.5 degrees.

Supplementary Figure S11

Subgroup analysis of NICU admission for studies with fever threshold of 38 degrees.

Supplementary Figure S12

Subgroup analysis of NICU admission for studies with vaginal delivery.

Supplementary Figure S13

Subgroup analysis of NICU admission for studies with epidural analgesia.

Supplementary Figure S14

Subgroup analysis of birth weight for studies with fever threshold of 37.5 degrees.

Supplementary Figure S15

Subgroup analysis of birth weight for studies with fever threshold of 38 degrees.

Supplementary Figure S16

Sensitivity analysis for infections and/or sepsis.

Supplementary Figure S17

Sensitivity analysis for NICU admission.

Supplementary Figure S18

Sensitivity analysis for birth weight.
