# Supplementary figures and images for: The effect of intrapartum maternal fever on neonatal outcomes: a systematic review and meta-analysis
Source: Front Pediatr. 2025 Sep 17;13:1571732. doi: 10.3389/fped.2025.1571732 (PMC12486603; doi:10.3389/fped.2025.1571732)

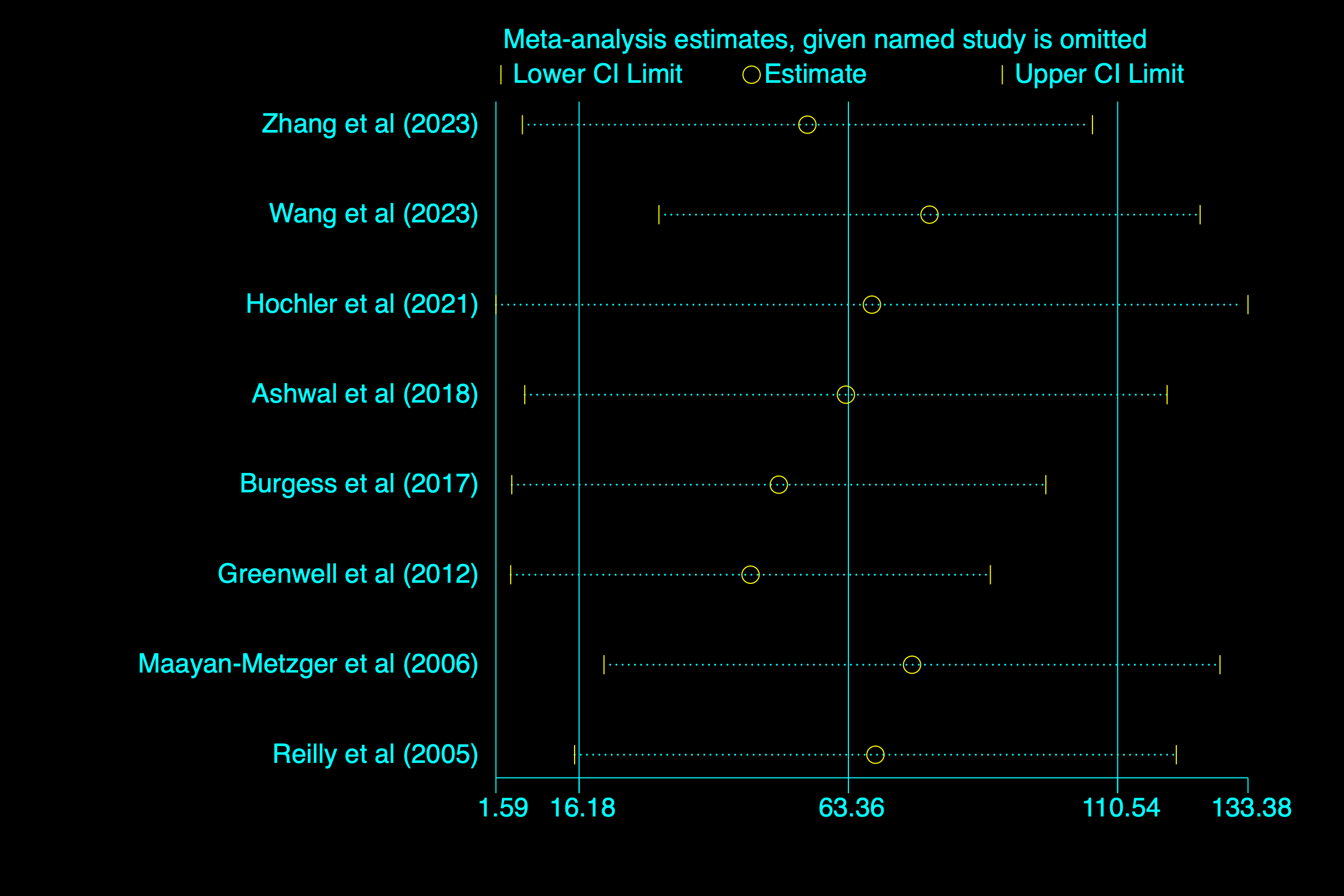

Supplement: Supplementary file 1 [file Datasheet1.zip › Supplementary Figure 18.jpg]

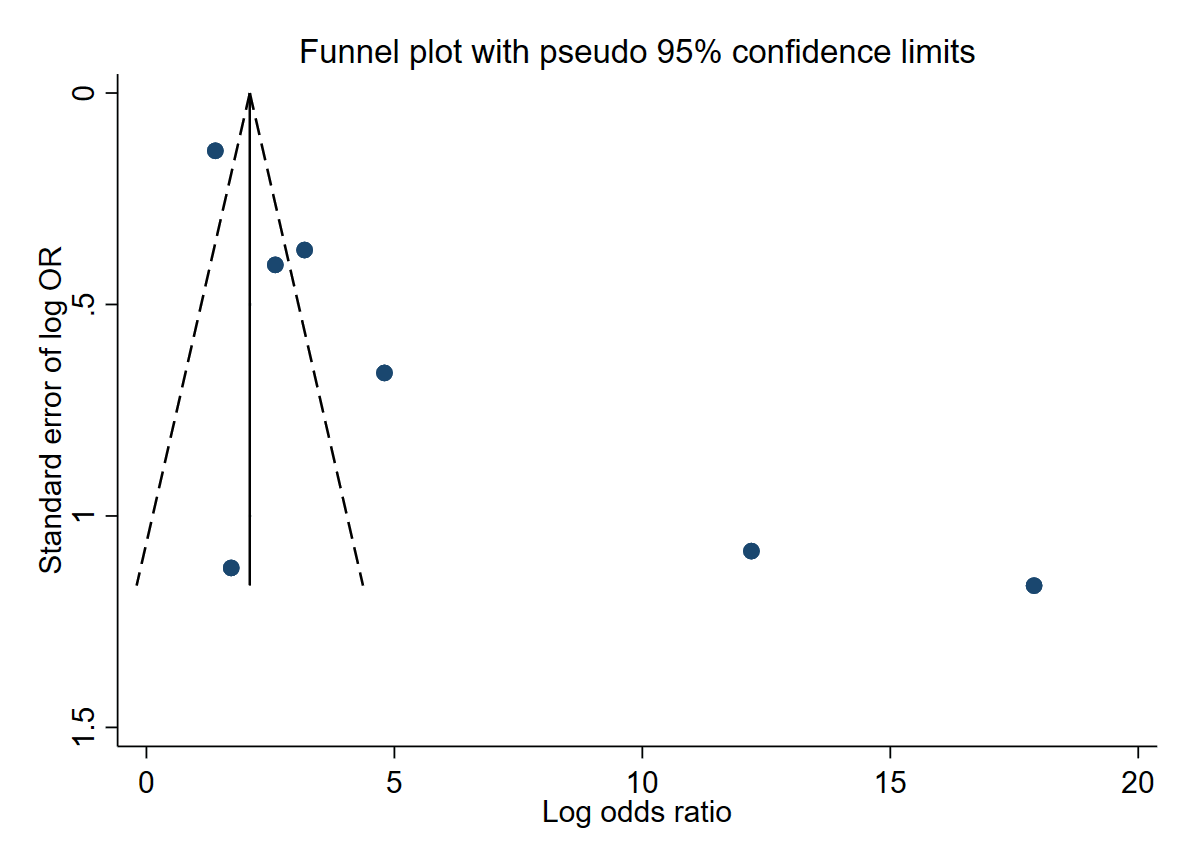

Supplement: Supplementary file 1 [file Datasheet1.zip › Supplementary Figure 1.tif]

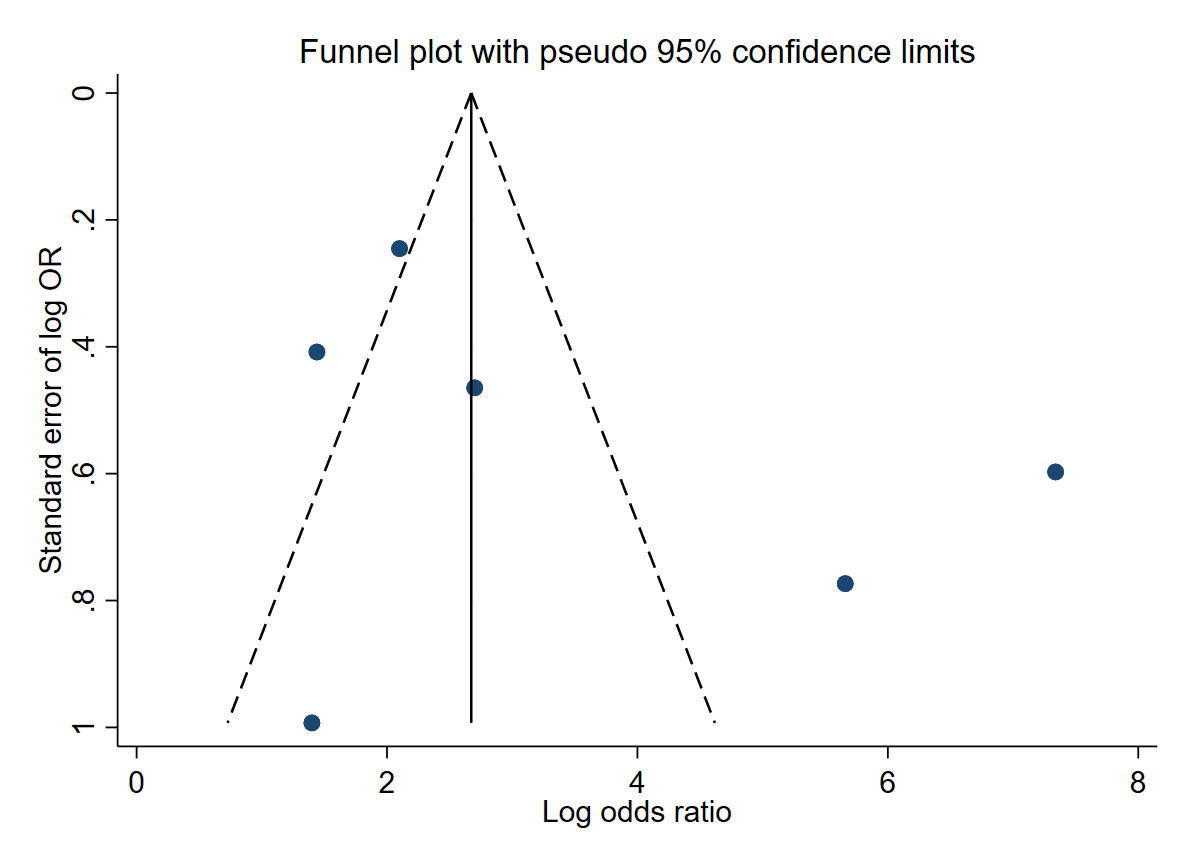

Supplement: Supplementary file 1 [file Datasheet1.zip › Supplementary Figure 2.tif]

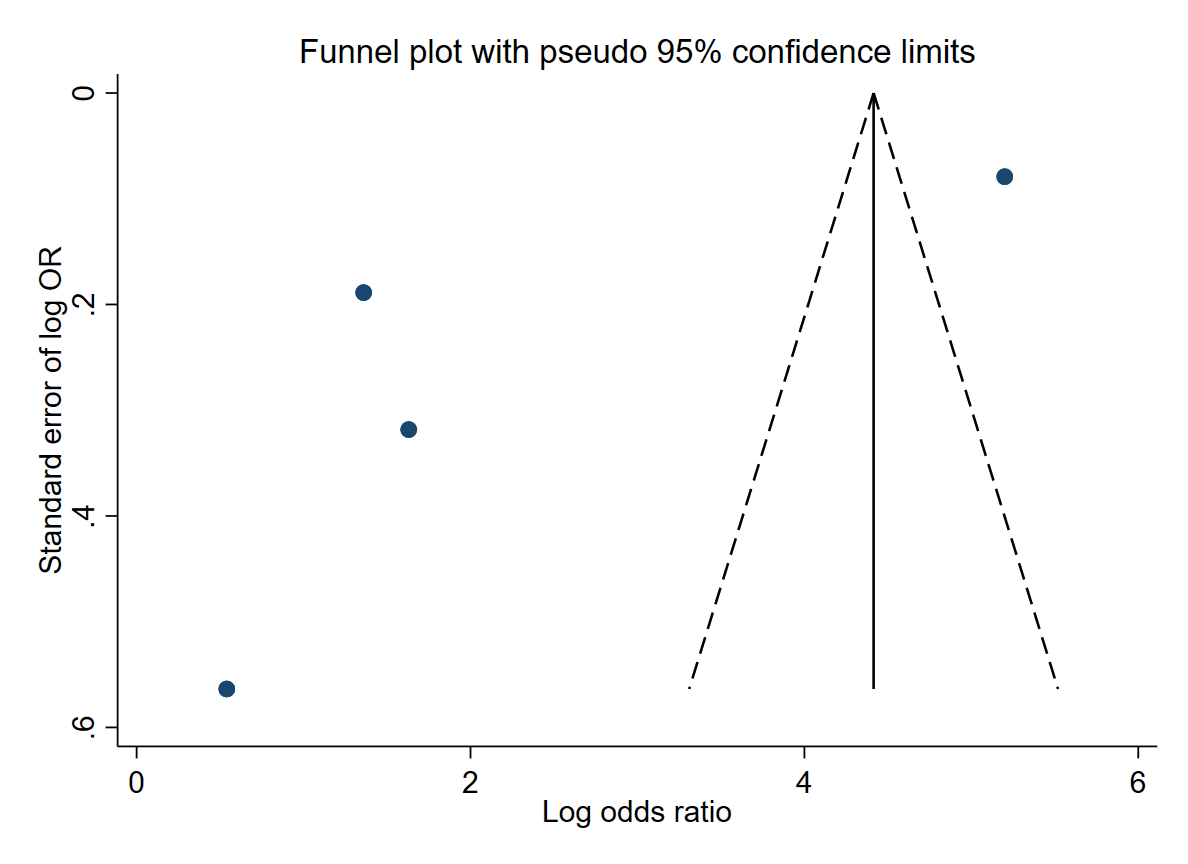

Supplement: Supplementary file 1 [file Datasheet1.zip › Supplementary Figure 3.tif]

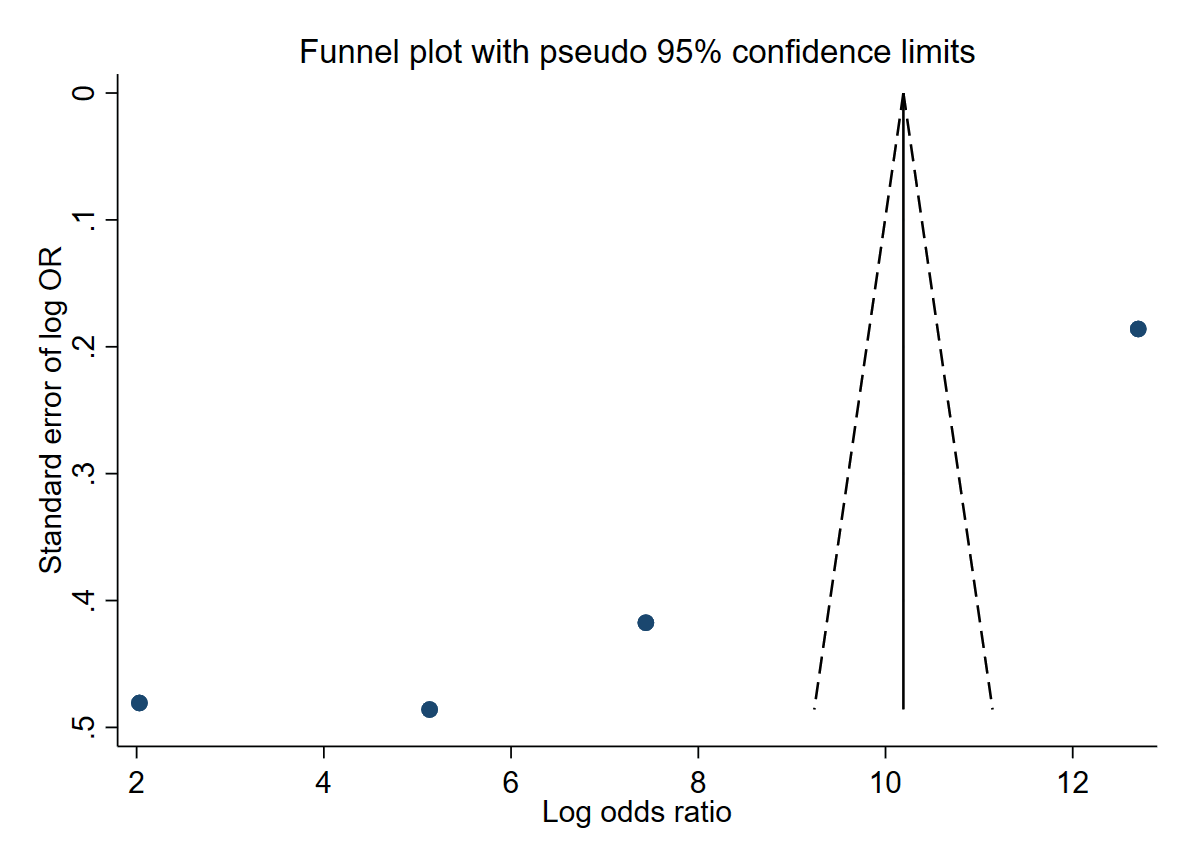

Supplement: Supplementary file 1 [file Datasheet1.zip › Supplementary Figure 4.tif]

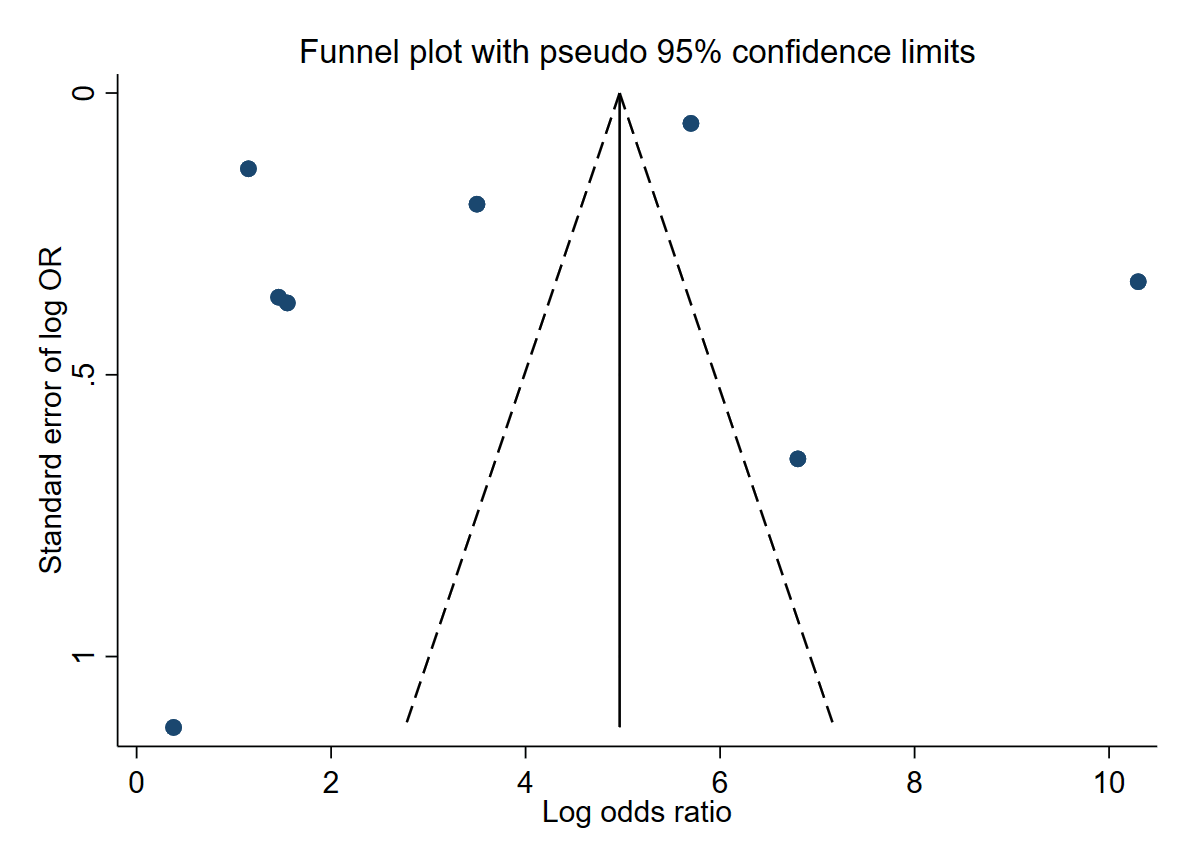

Supplement: Supplementary file 1 [file Datasheet1.zip › Supplementary Figure 5.tif]

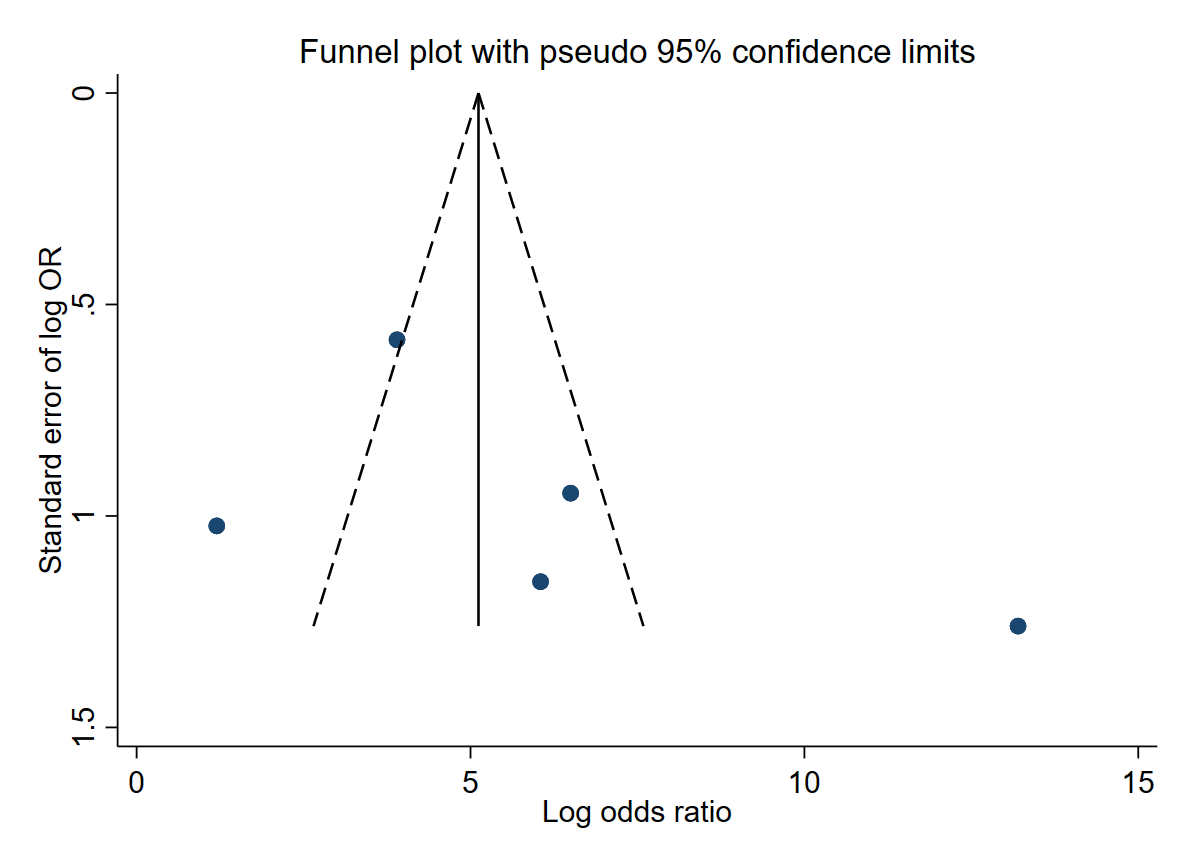

Supplement: Supplementary file 1 [file Datasheet1.zip › Supplementary Figure 6.tif]

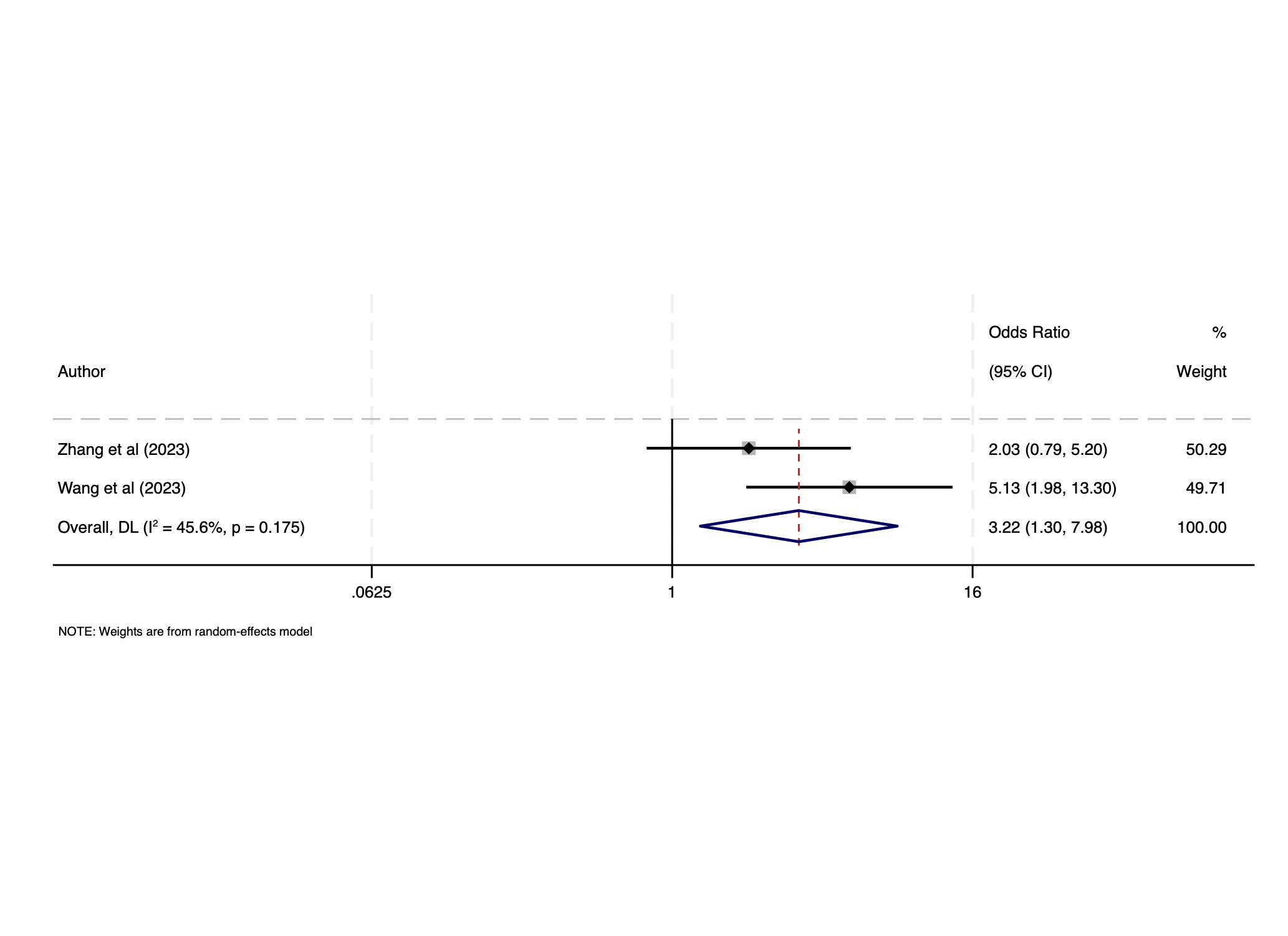

Supplement: Supplementary file 1 [file Datasheet1.zip › Supplementary Figure 7.jpg]

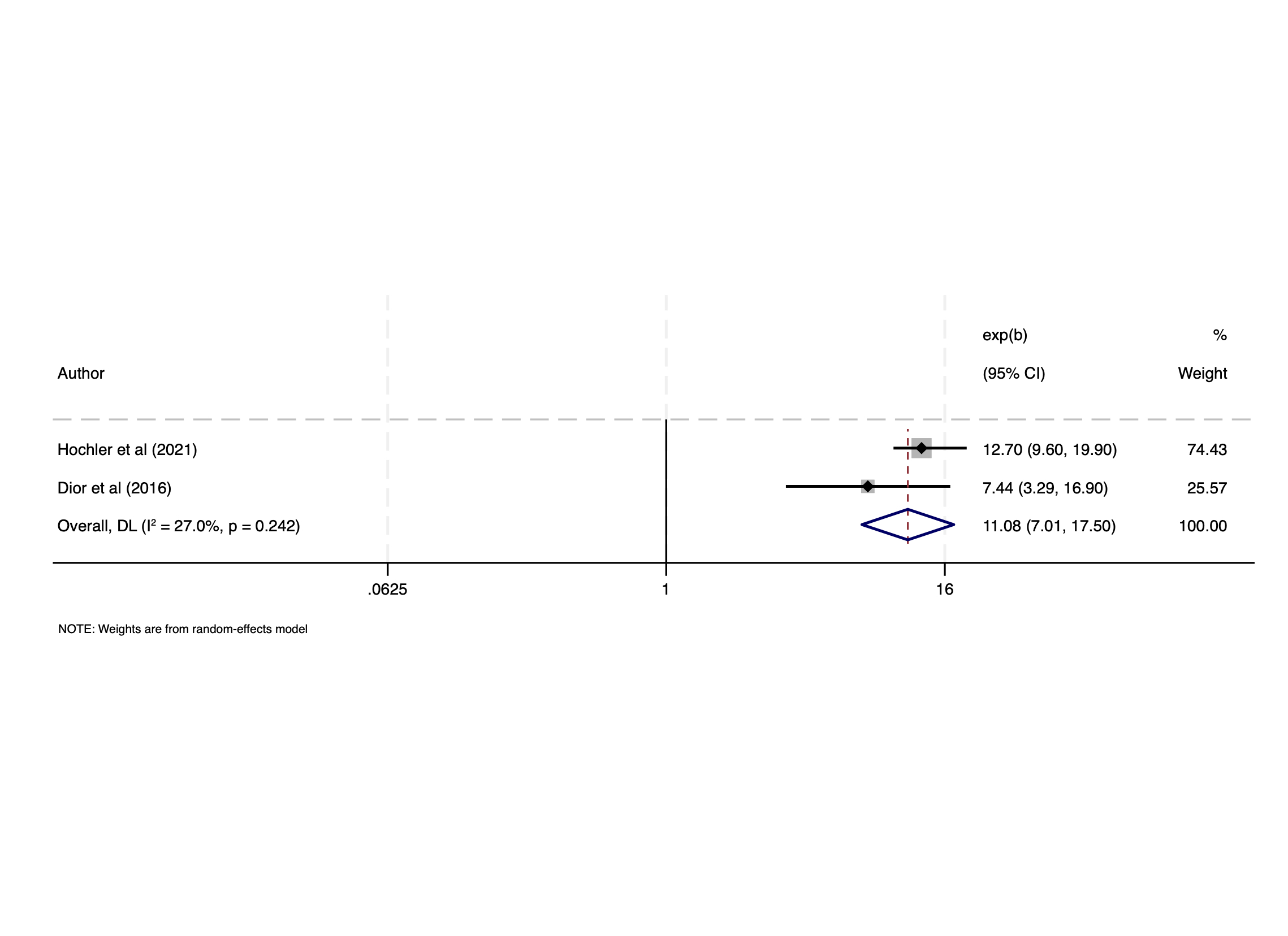

Supplement: Supplementary file 1 [file Datasheet1.zip › Supplementary Figure 8.jpg]

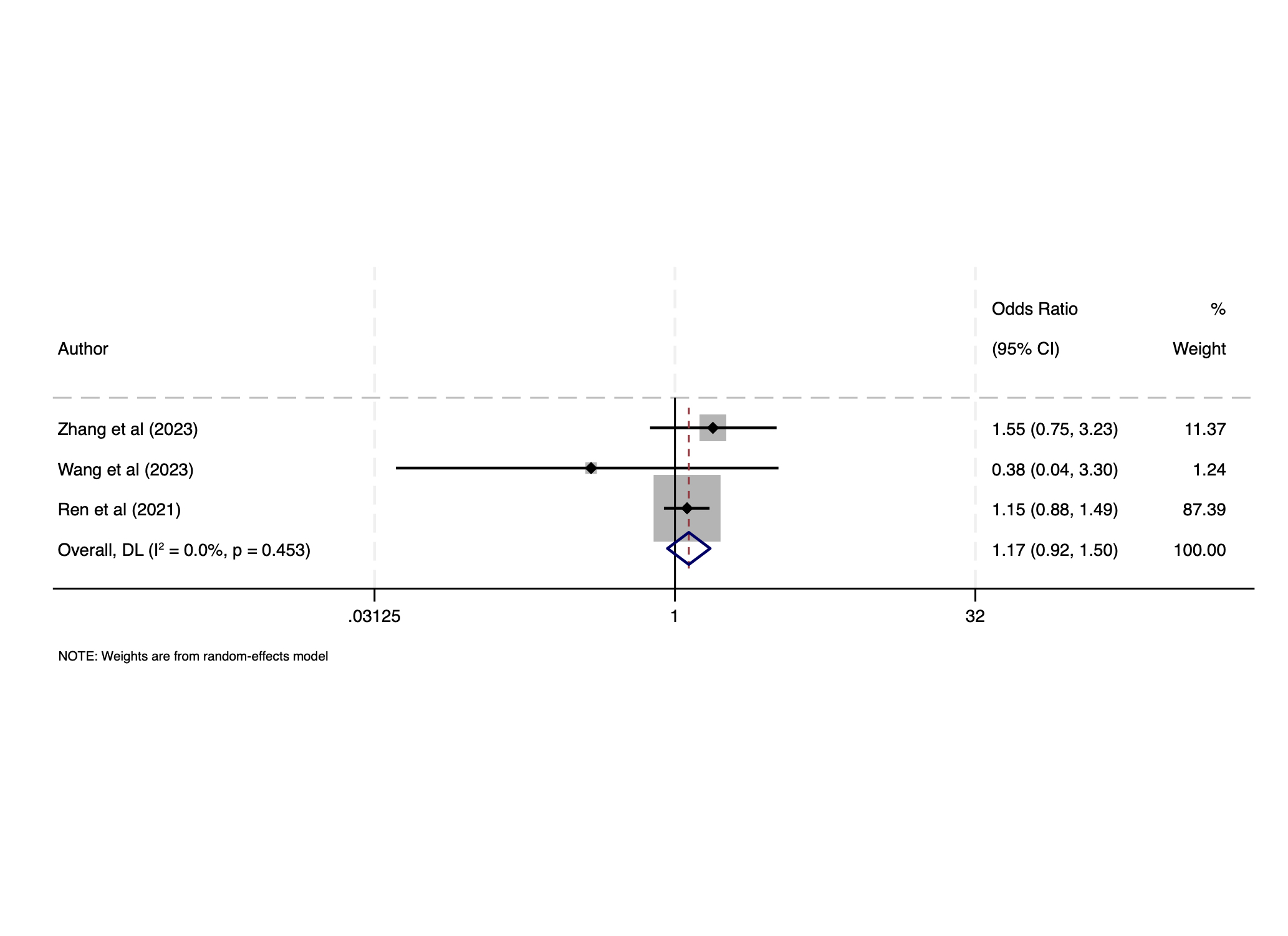

Supplement: Supplementary file 1 [file Datasheet1.zip › Supplementary Figure 10.jpg]

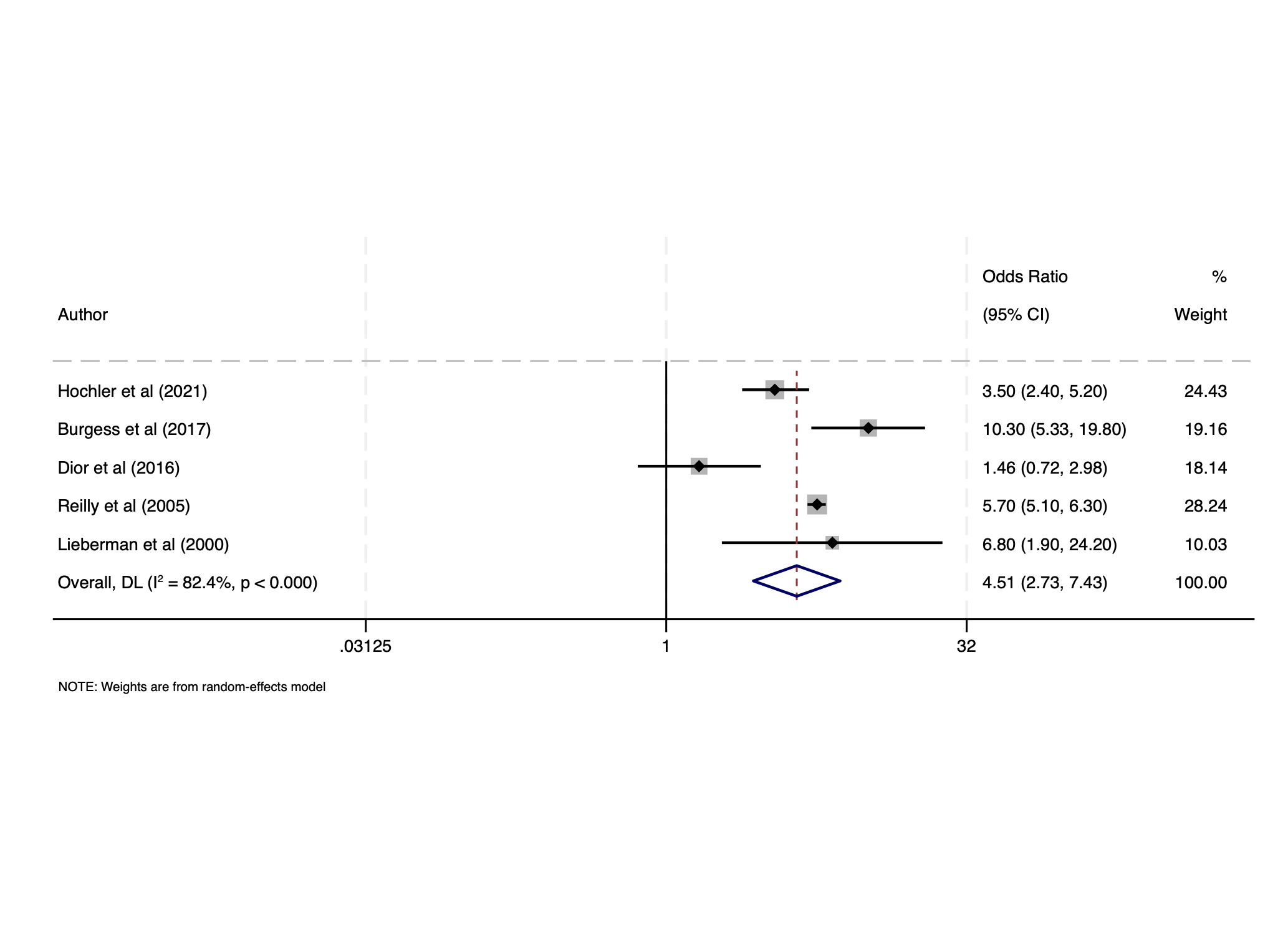

Supplement: Supplementary file 1 [file Datasheet1.zip › Supplementary Figure 11.jpg]

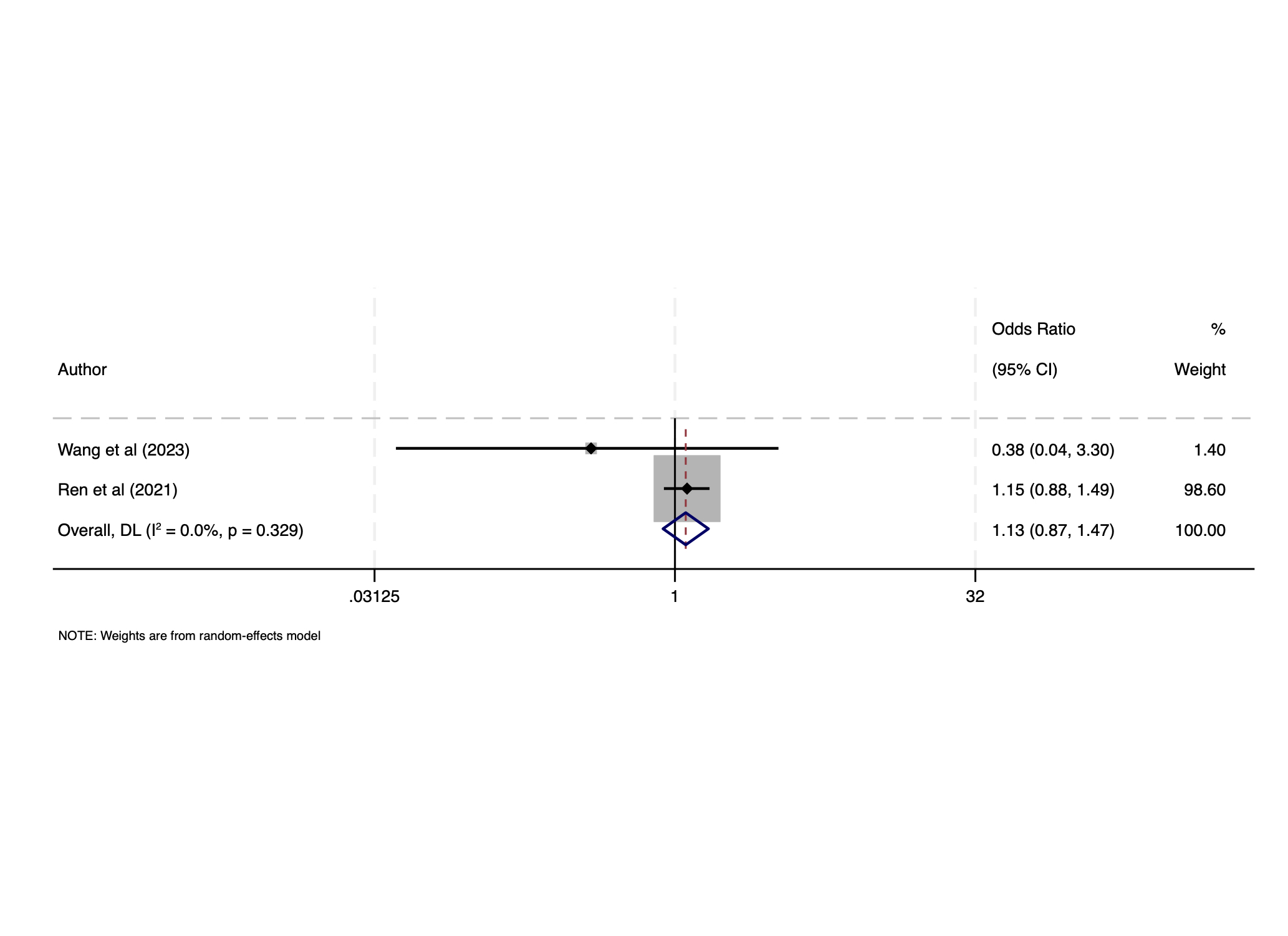

Supplement: Supplementary file 1 [file Datasheet1.zip › Supplementary Figure 13.jpg]

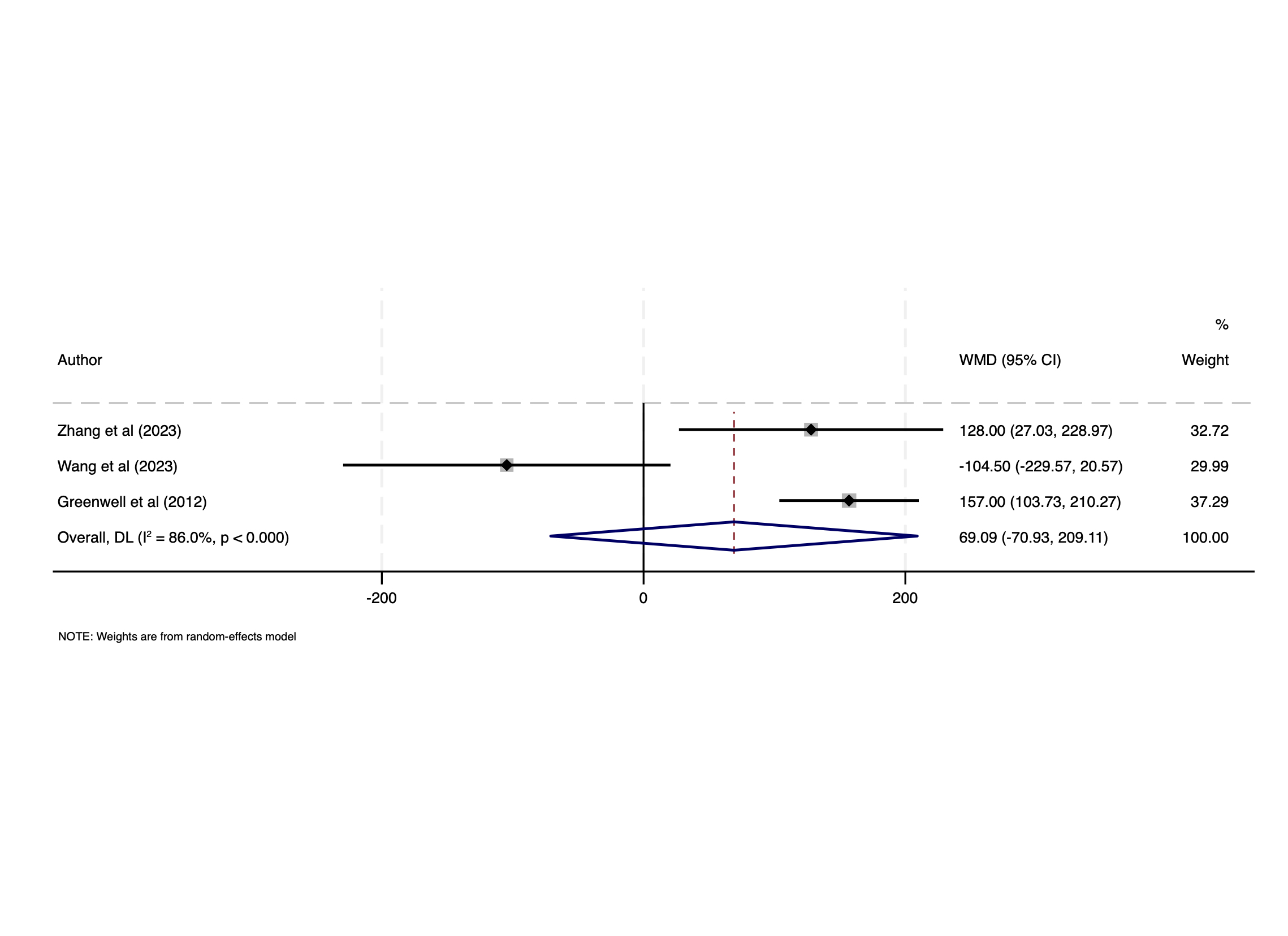

Supplement: Supplementary file 1 [file Datasheet1.zip › Supplementary Figure 14.jpg]

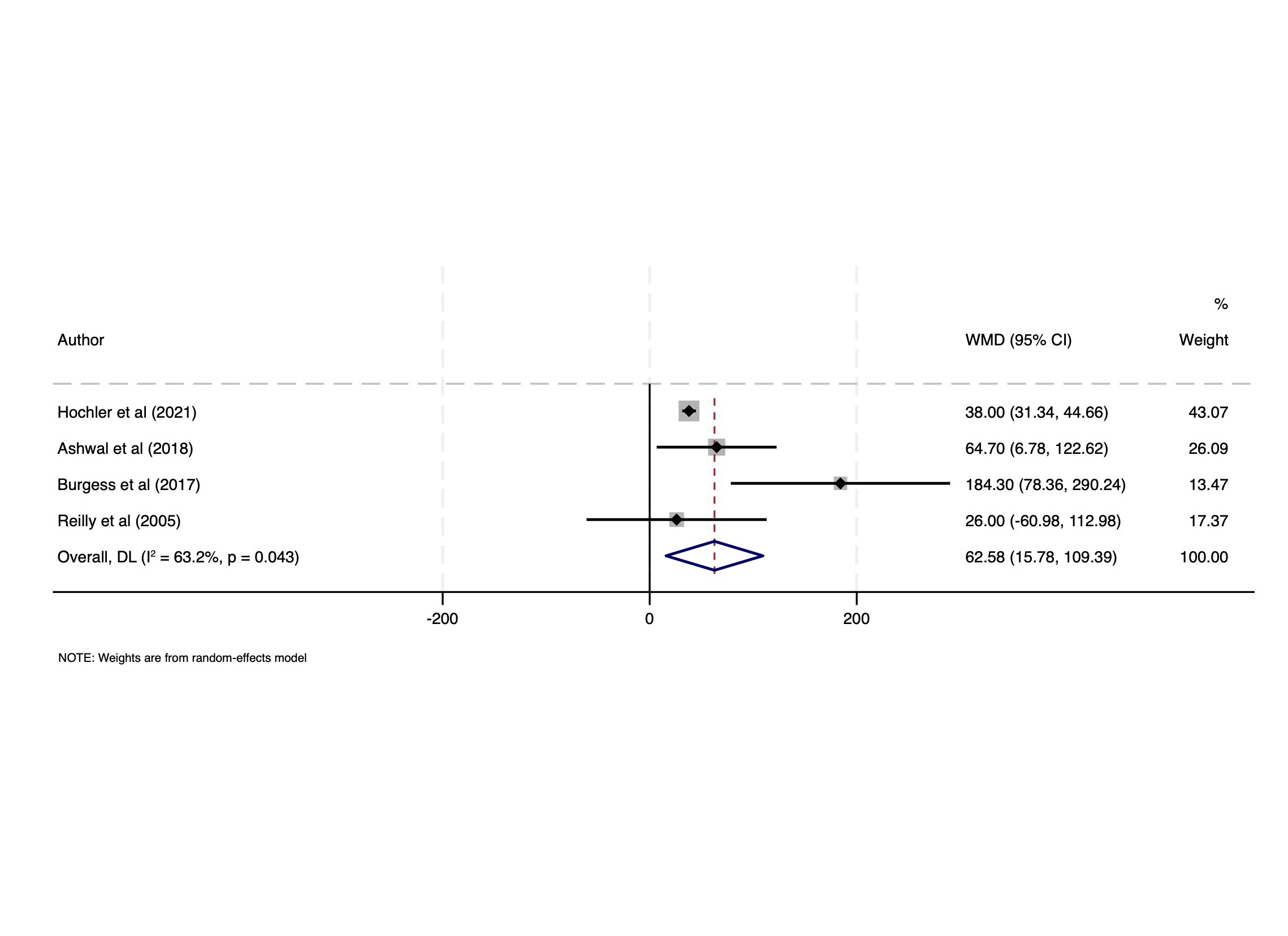

Supplement: Supplementary file 1 [file Datasheet1.zip › Supplementary Figure 15.jpg]

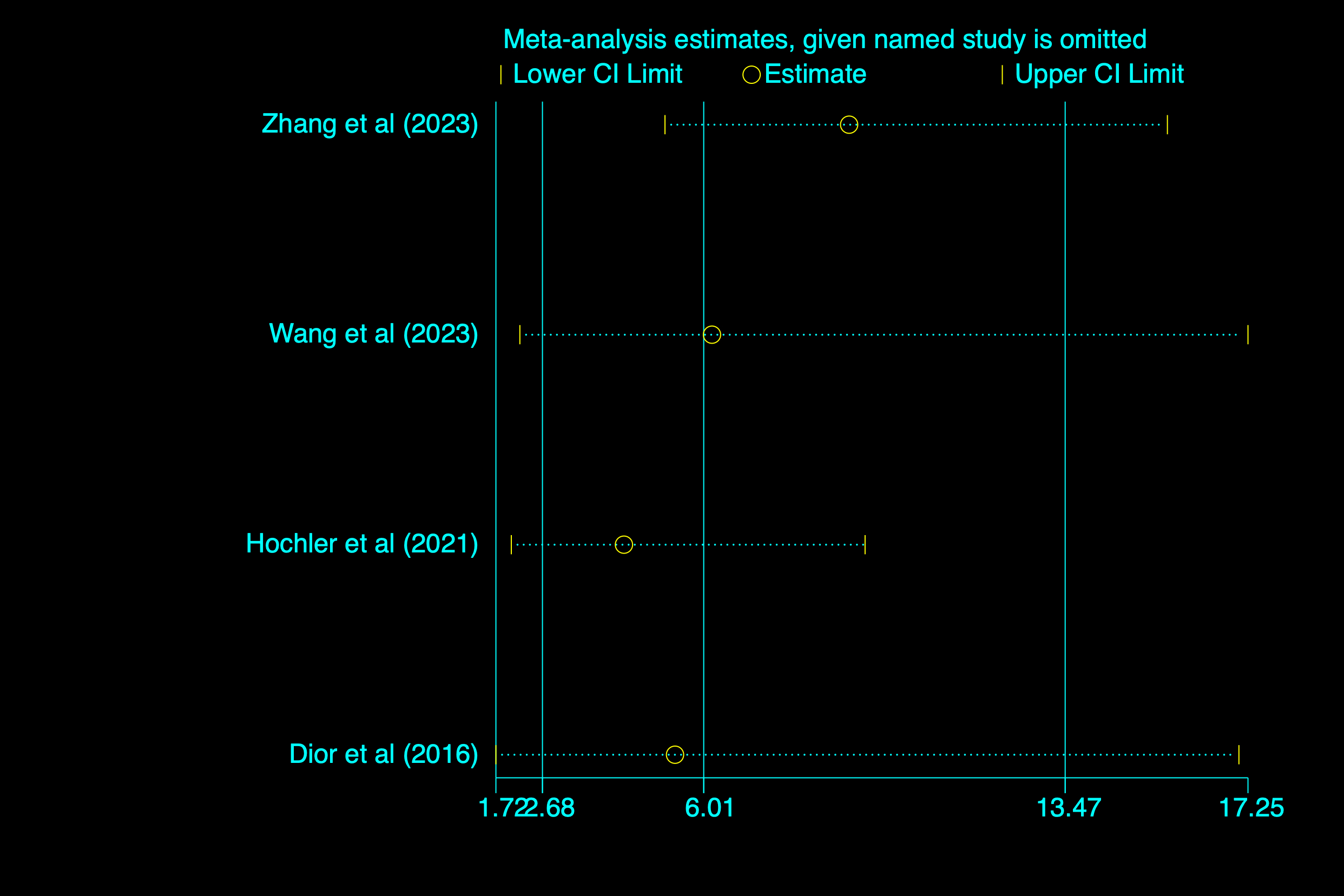

Supplement: Supplementary file 1 [file Datasheet1.zip › Supplementary Figure 16.jpg]

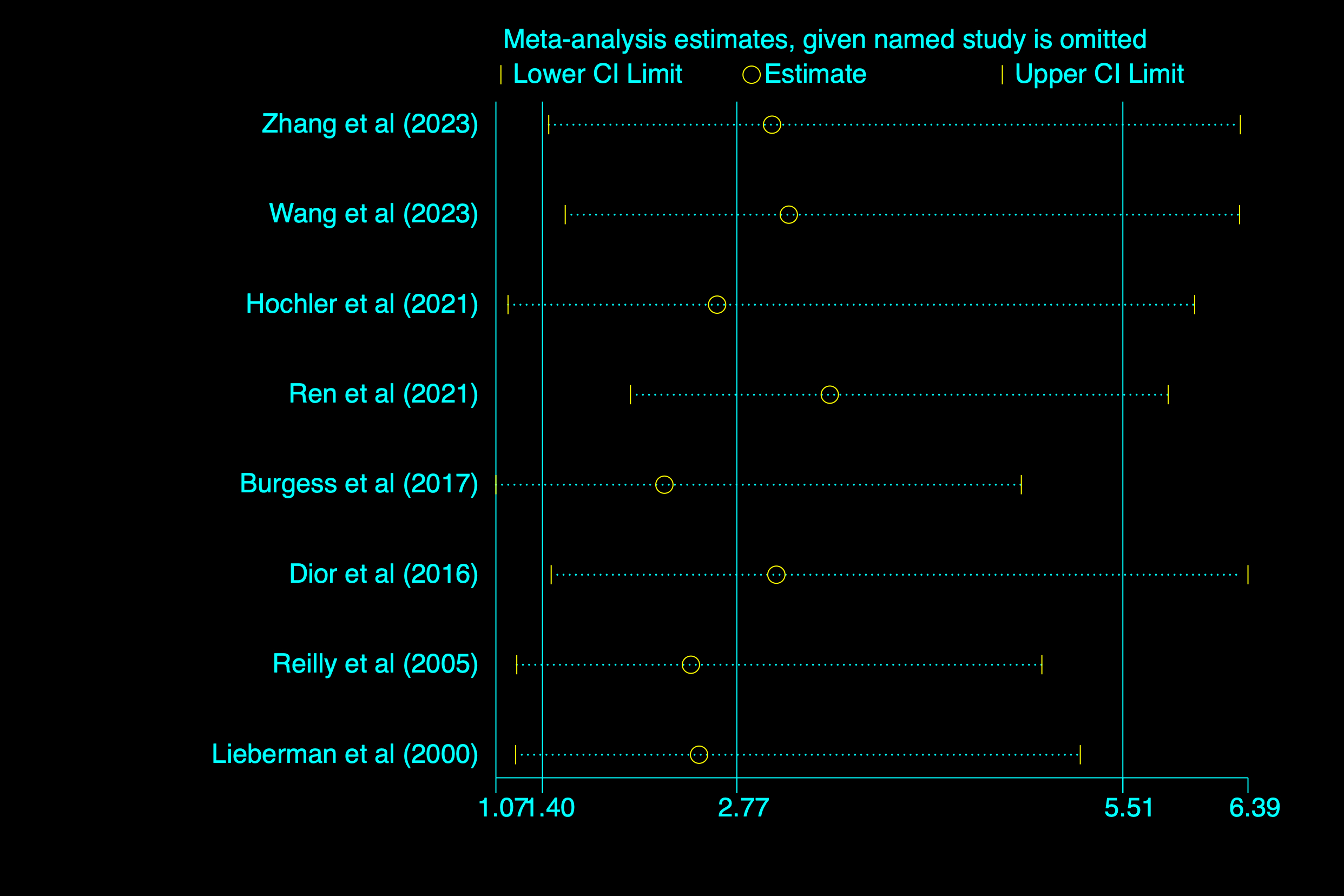

Supplement: Supplementary file 1 [file Datasheet1.zip › Supplementary Figure 17.jpg]
