# Supplementary material for: The effect of intrapartum maternal fever on neonatal outcomes: a systematic review and meta-analysis
Source: Front Pediatr. 2025 Sep 17;13:1571732. doi: 10.3389/fped.2025.1571732 (PMC12486603; doi:10.3389/fped.2025.1571732)
Supplement: Supplementary file 2 [file Datasheet2.pdf]

Supplementary Appendix:

Search Strategy:

PubMed:

(("intrapartum period"[MeSH Terms] OR intrapartum[Title/Abstract] OR "labor, obstetric"[MeSH Terms] OR labor[Title/Abstract] OR parturition[MeSH Terms] OR childbirth[Title/Abstract] OR "delivery, obstetric"[MeSH Terms] OR delivery[Title/Abstract])

AND (“fever”[MeSH Terms] AND maternal[Title/Abstract] OR "maternal fever"[Title/Abstract] OR "maternal hyperthermia"[MeSH Terms] OR "maternal hyperthermia"[Title/Abstract] OR "intrapartum fever"[Title/Abstract] OR "labor fever"[Title/Abstract] OR chorioamnionitis[MeSH Terms] OR chorioamnionitis[Title/Abstract] OR "intra-amniotic infection"[Title/Abstract] OR "intrauterine infection"[Title/Abstract])

AND ("neonatal outcome"[MeSH Terms] OR "neonatal outcomes"[Title/Abstract] OR "neonatal complication"[MeSH Terms] OR "neonatal complications"[Title/Abstract] OR "neonatal morbidity"[MeSH Terms] OR "neonatal morbidity"[Title/Abstract] OR "neonatal mortality"[MeSH Terms] OR "neonatal mortality"[Title/Abstract] OR "neonatal sepsis"[MeSH Terms] OR "neonatal sepsis"[Title/Abstract] OR "neonatal encephalopathy"[MeSH Terms] OR "neonatal encephalopathy"[Title/Abstract] OR "perinatal outcome"[Title/Abstract] OR "birth asphyxia"[MeSH Terms] OR "birth asphyxia"[Title/Abstract] OR "hypoxic ischemic encephalopathy"[MeSH Terms] OR "hypoxic ischemic encephalopathy"[Title/Abstract] OR Apgar score[MeSH Terms] OR Apgar[Title/Abstract] OR "respiratory distress syndrome"[MeSH Terms] OR "respiratory distress syndrome"[Title/Abstract] OR NICU[Title/Abstract] OR "neonatal intensive care"[Title/Abstract] OR seizure[MeSH Terms] OR seizure[Title/Abstract]))

Filters: Humans;

Scopus:

TITLE-ABS-KEY(intrapartum OR labour OR labor OR parturition OR childbirth OR “obstetric labor” OR “obstetric delivery”) AND TITLE-ABS-KEY(“maternal fever” OR “maternal hyperthermia” OR “intrapartum fever” OR “labor fever” OR chorioamnionitis OR “intra-amniotic infection” OR “intrauterine infection”) AND TITLE-ABS-KEY(“neonatal outcome” OR “neonatal outcomes” OR “neonatal complication” OR “neonatal complications” OR “neonatal morbidity” OR “neonatal mortality” OR “neonatal sepsis” OR “neonatal encephalopathy” OR

“perinatal outcome” OR “birth asphyxia” OR “hypoxic ischemic encephalopathy” OR Apgar OR “respiratory distress syndrome” OR NICU OR “neonatal intensive care” OR seizure)

EMBASE:

('intrapartum period'/exp OR intrapartum:ti,ab OR 'obstetric labour'/exp OR labour:ti,ab OR parturition:ti,ab OR 'childbirth'/exp OR childbirth:ti,ab OR 'obstetric delivery'/exp OR delivery:ti,ab)

AND ('maternal fever'/exp OR 'maternal fever':ti,ab OR 'maternal hyperthermia'/exp OR 'maternal hyperthermia':ti,ab OR 'intrapartum fever':ti,ab OR 'labor fever':ti,ab OR 'chorioamnionitis'/exp OR chorioamnionitis:ti,ab OR 'intra-amniotic infection':ti,ab OR 'intrauterine infection':ti,ab)

AND ('neonatal outcome'/exp OR 'neonatal outcome':ti,ab OR 'neonatal complication'/exp OR 'neonatal complication':ti,ab OR 'neonatal morbidity'/exp OR 'neonatal morbidity':ti,ab OR 'neonatal mortality'/exp OR 'neonatal mortality':ti,ab OR 'neonatal sepsis'/exp OR 'neonatal sepsis':ti,ab OR 'neonatal encephalopathy'/exp OR 'neonatal encephalopathy':ti,ab OR 'perinatal outcome':ti,ab OR 'birth asphyxia'/exp OR 'birth asphyxia':ti,ab OR 'hypoxic ischaemic encephalopathy'/exp OR 'hypoxic ischaemic encephalopathy':ti,ab OR 'apgar score'/exp OR apgar:ti,ab OR 'respiratory distress syndrome'/exp OR 'respiratory distress syndrome':ti,ab OR NICU:ti,ab OR 'neonatal intensive care unit'/exp OR 'seizure'/exp OR seizure:ti,ab)

Web of Science:

TS=(intrapartum OR labour OR labor OR parturition OR childbirth OR "obstetric delivery")

AND TS=("maternal fever" OR "maternal hyperthermia" OR "intrapartum fever" OR "labor fever" OR chorioamnionitis OR "intra-amniotic infection" OR "intrauterine infection")

AND TS=("neonatal outcome" OR "neonatal outcomes" OR "neonatal complication" OR "neonatal complications" OR "neonatal morbidity" OR "neonatal mortality" OR "neonatal sepsis" OR "neonatal encephalopathy" OR "perinatal outcome" OR "birth asphyxia" OR "hypoxic ischemic encephalopathy" OR Apgar OR NICU OR "respiratory distress syndrome" OR seizure)

Cochrane library:

(MeSH descriptor: [Intrapartum Period] explode all trees OR intrapartum:ti,ab,kw OR labour:ti,ab,kw OR labor:ti,ab,kw OR parturition:ti,ab,kw OR childbirth:ti,ab,kw OR "obstetric delivery":ti,ab,kw)

AND (MeSH descriptor: [Hyperthermia] explode all trees OR "maternal fever":ti,ab,kw OR "maternal hyperthermia":ti,ab,kw OR "intrapartum fever":ti,ab,kw OR "labor fever":ti,ab,kw OR chorioamnionitis:ti,ab,kw OR "intra-amniotic infection":ti,ab,kw OR "intrauterine infection":ti,ab,kw)

AND (MeSH descriptor: [Infant, Newborn] explode all trees OR MeSH descriptor: [Neonatal Death] explode all trees OR MeSH descriptor: [Sepsis, Neonatal] explode all trees OR MeSH descriptor: [Hypoxic-Ischemic Encephalopathy] explode all trees OR MeSH descriptor: [Respiratory Distress Syndrome, Newborn] explode all trees OR "neonatal outcome":ti,ab,kw OR "neonatal complication":ti,ab,kw OR Apgar:ti,ab,kw OR NICU:ti,ab,kw OR "perinatal outcome":ti,ab,kw OR "birth asphyxia":ti,ab,kw OR seizure:ti,ab,kw)

CNKI:

主题=(分娩期 OR 产程期 OR 阴道分娩 OR 剖宫产 OR 劳动)

AND 主题=(产妇发热 OR 分娩期发热 OR 劳动发热 OR 羊膜腔感染 OR 绒毛膜羊膜炎 OR 宫内感染)

AND 主题=(新生儿结局 OR 新生儿并发症 OR 新生儿发病率 OR 新生儿死亡 OR 新生儿感染 OR 新生儿窒息 OR Apgar OR 呼吸窘迫综合征 OR 缺氧缺血性脑病)
